# Analysis of the Distribution of Lymph Node Metastases and Their Impact on the Prognosis in Ductal Adenocarcinoma of the Distal Part of the Pancreas—A Single-Center Retrospective Study

**DOI:** 10.3390/cancers18121988

**Published:** 2026-06-18

**Authors:** Magdalena Gajda, Ewa Grudzińska, Sławomir Mrowiec

**Affiliations:** Department of Gastrointestinal Surgery, Medical University of Silesia, 40-752 Katowice, Poland; ewa.grudzinska@sum.edu.pl (E.G.); smrowiec@sum.edu.pl (S.M.)

**Keywords:** pancreatic ductal adenocarcinoma, PDAC, lymphadenectomy, distal pancreatectomy

## Abstract

**Background**: Lymphadenectomy is an important element of the surgical treatment of pancreatic ductal adenocarcinoma (PDAC). The study aimed to analyze the distribution of lymph node metastases in three nodal stations—group 10 (splenic hilum), group 11 (along the splenic artery), and group 18 (inferior pancreatic margin) depending on the location of PDAC in the distal pancreas: pancreatic neck (PN) vs. pancreatic body (PB) vs. pancreatic tail (PT). **Methods**: Data from 163 patients who underwent radical (R0) distal pancreatectomy with splenectomy (DP-S) with standard lymphadenectomy (removal of lymph nodes in groups 10, 11, and 18) for PDAC between 2010 and 2023 were analyzed. Patients were assigned to three groups based on the location of the primary tumor: neck (PN, *n* = 52), body (PB, *n* = 66), and tail (PT, *n* = 45). **Results**: The pattern of nodal metastasis was found to be dependent on the location of PDAC: group 10 (*p* < 0.001) is characteristic for tumor location in the PT (84.4%; 95% CI: 70–93%) and group 11 (*p* = 0.004) for tumors located in the PB (72.7%; 95% CI: 60–83%). Lymphatic drainage in group 18 constitutes a common zone, involved regardless of PDAC location (*p* = 0.185). In multivariate analysis, independent factors influencing overall survival were grading (HR 4.74; 95% CI 2.93–7.68; *p* < 0.001), lymph node metastasis in group 10 (HR = 3.65; 95% CI: 2.34–5.70; *p* < 0.001), group 11 (HR = 4.42; CI: 2.56–7.63; *p* < 0.001), group 18 (HR = 3.26; CI: 1.93–5.51; *p* < 0.001), and the use of neoadjuvant chemotherapy (HR = 0.57; CI: 0.39–0.84; *p* = 0.005). **Conclusions**: PDAC location in the distal pancreas influences metastasis to specific lymph node locations. Metastases to regional lymph node stations are associated with survival after DP-S procedures. In our retrospective cohort, neoadjuvant chemotherapy is associated with a lower risk of death and distant metastases due to PDAC after DP-S procedures.

## Simple Summary

The study aimed to analyze the distribution of lymph node metastases depending on the location of pancreatic cancer (PDAC) in the distal pancreas: neck vs. body vs. tail, in three nodal stations—group 10 (splenic hilum), group 11 (along the splenic artery), and group 18 (inferior pancreatic margin). The results suggest that PDAC location in the distal pancreas influences metastasis to specific lymph node locations. Metastases to regional lymph node stations are associated with survival after surgical resection. In this retrospective cohort, neoadjuvant chemotherapy was associated with a lower risk of death and distant metastases due to PDAC after resection procedures.

## 1. Introduction

Despite advances in diagnostic and therapeutic methods, pancreatic ductal adenocarcinoma (PDAC) remains one of the cancers with the poorest prognosis [1], with a 5-year overall survival rate of approximately 5–17% [2,3]. PDAC is predicted to become the second leading cause of cancer death by 2030 [4]. Although only 20% of newly diagnosed PDAC cases are eligible for surgical treatment [5], surgical removal of the tumor remains the only treatment option that offers a potential cure for PDAC [6]. The addition of chemotherapy to adjuvant treatment improves PDAC survival rates [3]. Early diagnosis of PDAC and synergistic treatment approaches [7] may also contribute to improving the prognosis of PDAC patients.

Lymphadenectomy is an important component of surgical treatment for PDAC. According to the International Study Group of Pancreatic Surgery (ISGPS) [8], surgery for PDAC requires the removal of at least 15 regional lymph nodes, which ensures local radicality, an acceptable risk of postoperative complications, and an adequate assessment of the disease stage. For PDAC located in the distal part of the pancreas, standard lymphadenectomy removes lymph nodes at three anatomical locations—group 10 (splenic hilum), group 11 (along the splenic artery), and group 18 (inferior pancreatic margin) [8].

However, in patients undergoing distal pancreatectomy, the location of PDAC can vary greatly—from tumors located just next to the pancreatic head, in the neck of the pancreas, through tumors of the body, to tumors in the tail of the pancreas, adjacent to the spleen. This different location may also be associated with differences in lymphatic drainage [9].

This study aimed to analyze the distribution of PDAC lymph node metastases groups 10, 11, and 18, depending on the location of PDAC in the distal part of the pancreas (neck vs. body vs. tail). Furthermore, independent prognostic factors for survival were identified: overall survival (OS), local recurrence-free survival (PFS-LR), and distant metastasis-free survival (PFS-DM).

## 2. Materials and Methods

### 2.1. Screening for the Study and Characteristics of the Study Cohort

**Figure 1:**
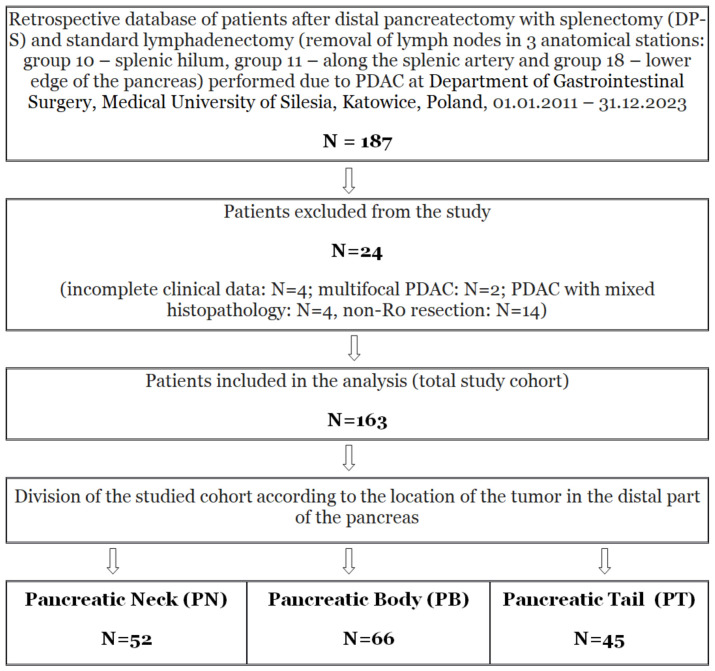
Flowchart showing patient screening for the study.

**Figure 2:**
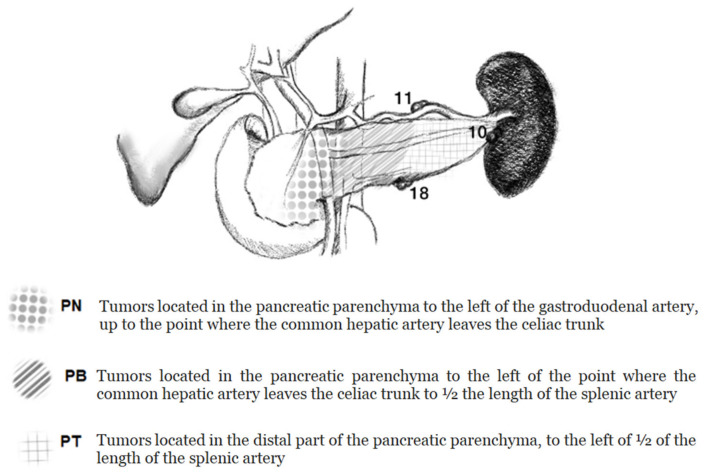
The pancreatic neck (PN), pancreatic body (PB), and pancreatic tail (PT), with marked location of nodal groups: group 10 (splenic hilum), group 11 (all lymph nodes along the splenic artery, without distinction of the proximal and distal parts), and group 18 (inferior margin of the pancreas).

The study analyzed data of 163 patients (Figure 1), who were assigned to three groups based on the location of the primary tumor: neck (*n* = 52), body (*n* = 66), and tail (*n* = 45) (Figure 2). Qualification for surgery and the location of PDAC was determined based on preoperative computed tomography (CT) of the abdomen and pelvis with oral and intravenous contrast, and verified intraoperatively. Each patient underwent a distal pancreatic resection with splenectomy (DP-S) with standard lymphadenectomy according to ISGPS criteria [8], i.e., removal of the lymph node groups 10, 11, and 18. The nodal groups were analyzed without differentiating the proximal and distal parts.

The assessment of resectability was conducted based on the National Comprehensive Cancer Network (NCCN) criteria [10]. Patients with potentially resectable PDAC (PR-PDAC: no contact with arteries such as celiac trunk (CeT), superior mesenteric artery (SMA), common hepatic artery (CHA); no contact with the superior mesenteric vein (SMV) and portal vein (PV) or adhesion to ≤180 degrees of the circumference without alteration of the vein’s shape) were eligible for upfront surgery. Patients with locally advanced PDAC (LA-PDAC: distant metastases, including metastases to non-regional lymph nodes; tumor abuts > 180 degrees of the circumference of the SMA or CeT; tumor abuts the CeT or invades the aorta; SMV/PV reconstruction is not possible due to invasion or lumen occlusion, which may be caused by the tumor or a thrombus) or borderline resectable PDAC (BR-PDAC: tumor abuts the CeT ≤ 180 degrees of the circumference; tumor abuts the CeT > 180 degrees of the circumference without aortic involvement and an intact, uninvolved gastroduodenal artery, thus allowing for a modified Appleby procedure; tumor abuts the SMV or PV > 180 degrees of the circumference, abutment ≤ 180 degrees with deformation of the vessel or thrombosis in the vein but with normal vessels proximal and distal to the site of invasion, which allows for safe and radical resection with reconstruction; tumor abuts the inferior vena cava (IVC)) received neoadjuvant chemotherapy (6–8 cycles of the FOLFIRINOX regimen). After 6–8 cycles of chemotherapy, a follow-up CT was performed—patients with a tumor assessed as PR-PDAC or BR-PDAC were qualified for surgery. Patients with LA-PDAC or systemic dissemination after neoadjuvant chemotherapy were not eligible for surgery.

Only patients after R0 resection—tumor distance from surgical margin > 1 mm—were included in the study. We excluded a total of 14 patients who had R2 resection (macroscopically visible cancer) in whom the resection was palliative, protecting from developing obstruction, and performed as an emergency rather than a procedure with the intention to treat.

After surgery, all patients in our cohort received adjuvant chemotherapy (6–12 cycles of the FOLFIRINOX regimen). No other systemic treatment was administered.

Tumor staging was performed using the TNM scale. The pathological examination of the specimens was carried out by two independent, experienced pathologists.

Postoperative follow-up was performed by the operating surgeon or an oncologist. The entire study cohort was followed according to our institution’s standard. Follow-up included CT scans of the abdomen and pelvis at 6-month intervals for the first 2 years, followed by 12-month intervals for the next 3 years, and 12-month or 24-month intervals for the rest of the patient’s life. CT scan results were analyzed by two experienced radiologists for local recurrence (in the pancreatic stump, postoperative site, or regional lymph nodes) and distant metastases (liver, peritoneum, lungs, bones, adrenal glands, bladder, extraregional lymph nodes).

The study analyzed the following endpoints: OS—time from surgery to death; PFS-LR—time from surgery to local recurrence; and PFS-DM—time from surgery to distant metastases. The variables included in the multivariate analysis were demographic (age, gender, BMI), tumor characteristics (tumor size, grading G1–G4, pT category, resectability), neoadjuvant chemotherapy, pathological examination results (perineural invasion—PNI, vascular invasion—VI) and nodal parameters (number of removed lymph nodes, number of nodes with metastases, ratio of lymph nodes with PDAC metastases to the total number of removed lymph nodes—lymph node ratio—LNR, metastatic status per anatomical station N+ vs. N− for groups 10, 11 and 18).

Our work is a retrospective analysis based solely on existing, fully anonymized data, with no patient involvement. As such, in accordance with the national legislation, it is not considered a medical experiment, and the consent of the bioethics committee and the patient’s consent are not required.

### 2.2. Statistical Analysis

The level of statistical significance was set at α = 0.05 for all analyses. To reduce the risk of type I error in multiple comparisons, a Benjamini–Hochberg false discovery rate (FDR) correction of 0.05 was applied. This correction was applied to both descriptive analyses (pairwise comparisons—Supplementary Materials, Section S1, Tables S1–S3) and univariate Cox analyses (adjusting the *p*-value per endpoint).

Analyses were designated a priori as confirmatory or exploratory. The confirmatory analyses comprised the association between tumor location and nodal involvement at stations 10, 11, and 18, and the multivariable prognostic models. The baseline comparisons, the univariate screening, the data-driven determination of the LNR cut-point, and all pairwise post hoc contrasts were exploratory and hypothesis-generating. Benjamini–Hochberg FDR control (q = 0.05) was applied within each family of tests, separately per endpoint. *p*-values are descriptive and do not inform model specification.

Characteristics of the study population were presented in STROBE format, stratifying data by tumor location. Continuous variables were described using the median with interquartile range (IQR: Q1–Q3). Categorical variables were expressed as absolute counts with percentages. 95% confidence intervals were determined using Student’s *t*-test for continuous variables and the Wilson method for categorical variables. The Kruskal–Wallis test for continuous variables (due to non-normal distributions) and the two-sided Fisher’s exact test for categorical variables were used to compare the three groups (PN vs. PB vs. PT). When global significance was achieved, post hoc analyses were performed: Dunn’s test for pairwise comparisons of continuous variables and Fisher’s exact test for categorical variables, with FDR correction. The strength of the association was assessed using Cramér’s V with 95% CI. The correlation between the number of examined lymph nodes and the number of nodes with metastases was assessed using the Spearman rank correlation coefficient (ρ), with a 95% CI determined using the BCa bootstrap method (bias-corrected and accelerated).

The optimal cutoff for lymph node ratio (LNR) was identified exploratorily (data-driven—the obtained thresholds require validation in an independent cohort) using the standardized maximum statistics test with approximation of the *p*-value distribution. The analysis was performed separately for each endpoint (OS, PFS-LR, PFS-DM).

To reduce the confounding effect resulting from the uneven distribution of prognostic variables between location groups, entropy balancing was used—a method of weighting observations by inverse probability weighting (IPW). Entropy balancing is a nonparametric reweighting method that assigns weights that ensure precise equalization of the moments of covariate distributions between groups, within the framework of the average treatment effect (ATE) estimate (Supplementary Materials, Section S3, Table S5). The balancing procedure included 11 covariates selected a priori based on their documented prognostic significance in the PDAC literature: age, sex, BMI, tumor size, grading, pT category, resectability, neoadjuvant chemotherapy, preoperative CA 19-9 level, perineural invasion (PNI), and vascular invasion (VI). The entropy balancing procedure achieved its initial goal—full equalization of the 11 covariates between the three nonrandomized tumor site groups, while maintaining an effective sample size that allowed for further multivariate modeling (Supplementary Materials, Section S3, Figure S1). The resulting weights form the foundation for all subsequent survival analyses: Kaplan–Meier estimation (Section 3.4) and univariate and multivariate Cox models (Section 3.5 and Section 3.6), ensuring that the location effects observed in these analyses reflect—within the limits of measurable variables—the true effect of tumor position and not an artifact of baseline differences between groups.

The survival function was estimated using the Kaplan–Meier method, separately for each time point and stratification (N+/N− status per nodal station). Estimation was performed with entropy balancing weights. 95% confidence intervals were determined using the Greenwood method. Comparisons between survival curves were performed using the global log-rank test (three groups) and pairwise FDR-corrected. Median survival with 95% CI and 1-, 2-, and 3-year survival probabilities were reported from the weighted estimates.

Separate univariate and multivariate Cox models, with entropy-balancing weights, were fitted for each predictor. Results were expressed as hazard ratios (HRs) with 95% CIs. *p*-values were adjusted using the FDR method for each endpoint. Variables with a *p*-value < 0.20 from the univariate analysis were considered candidates for the multivariate model. The final models for the multivariate analysis were selected using backward elimination. Results were reported as adjusted HRs with 95% CIs.

Grading was analyzed as a binary variable (well or moderately differentiated, G1–2, versus poorly differentiated or undifferentiated, G3–4); the single G4 case was combined with G3. This avoided unstable estimation arising from the sparsely populated G4 category (*n* = 1).

The proportional-hazards assumption was examined for each covariate and globally using scaled Schoenfeld residuals. Where the assumption was not met, the corresponding hazard ratio is interpreted as an effect averaged over the observation period, and a time-interaction sensitivity model was fitted to characterize the direction of the departure. (Supplementary Materials, Section S5, Table S7).

Internal validation. Discrimination of the multivariable models was summarized by Harrell’s concordance index and corrected for optimism using 1000 bootstrap resamples, with covariate selection repeated within each resample. Calibration was evaluated by the bootstrap optimism-corrected calibration slope, a value near 1 denoting agreement between predicted and observed risk and the absence of material overfitting. (Supplementary Materials, Section S4, Table S6 and Section S6, Table S8).

All calculations were performed using R version 4.5.2 (R Core Team, Vienna, Austria), running on Windows 10 Pro 64-bit (Microsoft Corporation, Redmond, WA, USA).

## 3. Results

### 3.1. Clinicopathological and Demographic Characteristics of the Study Population by Tumor Location

The study groups were comparable in terms of demographic characteristics and clinical parameters (Table 1). The median age for the entire cohort was 67.0 years (IQR: 60.0–70.0), and the gender distribution was nearly symmetrical (women 50.9%; men 49.1%), with no significant differences between groups (*p* = 0.229, *p* = 0.960). Body mass index, smoking, ASA, and the frequency of neoadjuvant chemotherapy did not differ significantly between groups.

However, a distinctly different structure was revealed in terms of tumor characteristics. Tumor location in PT emerged as a factor associated with a more advanced stage of disease. Tumor size was one of the most significant differentiating variables in the analysis (*p* < 0.001): the median tumor size in PT was 4.0 cm (IQR: 3.0–4.0), exceeding both PN (median 2.5 cm) and PB (median 3.0 cm). This size gradient directly translated into the distribution of pT categories (*p* < 0.001): in PT, pT2 (64.4%) and pT3 (24.4%) tumors predominated, while in PN, almost half of the cases (46.2%) were pT1 tumors—illustrating the local advancement gradient increasing from the proximal to the distal part of the pancreas. The staging distribution followed the same trend: the proportion of patients with stage III increased from 32.7% in PN, through 40.9% in PB, to 53.3% in PT (*p* = 0.035). The pN category revealed a similar pattern—pN0 was found in 46.2% of patients with PN but only in 15.6% of patients with PT, while the proportion of pN2 increased in the opposite direction, from 32.7% to 53.3% (*p* = 0.025).

Potential confounding variables did not exhibit comparable disparities. Histological grading was homogeneous across all three groups (*p* = 0.992), with moderately differentiated G2 tumors predominating (62.6%), and resectability (resectable vs. borderline) did not reveal significant disparities (*p* = 0.814). Histopathological aggressiveness features—perineural invasion (PNI; 73.0% in the entire cohort) and vascular invasion (VI; 62.6%)—were relatively evenly distributed, although PNI showed a trend toward a higher incidence in PB (81.8% vs. 63.5% in the PN; *p* = 0.075), which, however, did not reach significance after adjustment.

The distribution of postoperative complications according to the Clavien–Dindo classification did not differ significantly (*p* = 0.479).

The total number of examined lymph nodes did not differ significantly between groups (*p* = 0.085), confirming a comparable extent of lymphadenectomy regardless of tumor location (Table 2). However, the total number of nodes with confirmed metastases showed significant differences (*p* = 0.013): the median in PN was 1.0 (IQR: 0.0–5.5), in PB was 3.0 (IQR: 0.0–8.0), and in PT was 4.0 (IQR: 1.0–7.0). LNR increased from a median of 0.0 in the PN to 0.2 in PT (*p* = 0.022).

Median OS in PT was 20.0 months (IQR: 12.0–32.0) versus 29.0 months in PN and 24.0 months in PB (*p* = 0.102. Distant metastases occurred in 68.7% of patients in the entire cohort, with the proportion in PN (55.8%) being lower than in PB (77.3%) and PT (71.1%; *p* = 0.041). Local recurrence did not show any significant difference (*p* = 0.597).

The number of events per variable in the final models was 25.2 (OS, 126 events), 16.0 (PFS-DM, 112 events), and 13.0 (PFS-LR, 52 events), each exceeding the conventional minimum of ten. Discrimination, corrected for optimism by 1000 bootstrap resamples, was 0.831 (OS), 0.828 (PFS-DM), and 0.803 (PFS-LR), with optimism below 0.01 in every model, indicating the parsimonious models are not materially overfit (Supplementary Materials, Section S4, Table S6).

Division into individual nodal stations revealed a characteristic pattern of metastasis (Table 2). In group 10, the difference was extremely large (*p* < 0.001): metastases were present in 84.4% (95% CI: 70–93%) of patients with PT, compared to only 5.8% (CI: 1.5–17%) in PN and 10.6% (CI: 4.7–21%) in PB. In group 11, the hierarchy was reversed: the highest percentage of N+ was observed in PB—72.7% (CI: 60–83%), compared to 53.8% in PN and 42.2% in PT (*p* = 0.004). In group 18, no significant intergroup differences were found (*p* = 0.185): metastases were distributed relatively evenly (from 42.3% in PN to 59.1% in PB).

### 3.2. Distribution of Regional Lymph Node Metastases in Groups 10, 11, and 18 by Tumor Location

**Figure 3:**
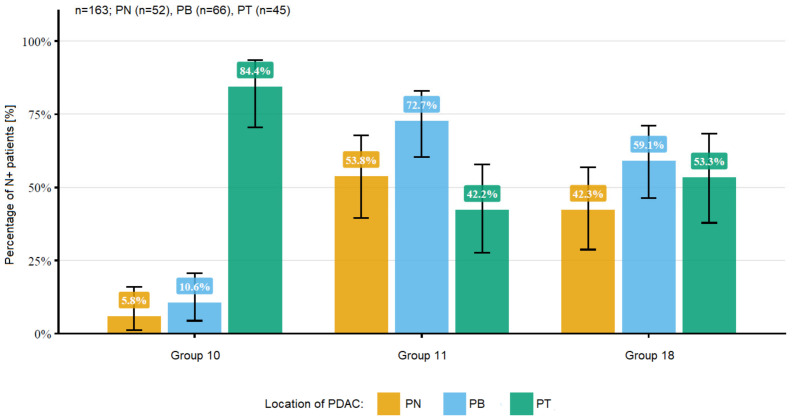
Incidence of lymph node metastases (N+) by anatomical station and tumor location. PN = pancreatic neck; PB = pancreatic body; PT = pancreatic tail.

The strength of the association between the frequency of nodal metastases in individual stations and tumor location was measured using Cramér’s V coefficient (Table 3, Figure 3).

The frequency of metastases in group 10 reached 84.4% of patients with PT, compared to 10.6% in PB and only 5.8% in PN (*p* < 0.001; Table 3, Figure 3). Cramér’s V coefficient in group 10 was 0.75 (95% CI: 0.64–0.86), indicating a strong effect, close to full determination. Pairwise analyses (Supplementary Materials, Section S1, Tables S1–S3) confirmed that this effect was solely due to pairs involving pancreatic tail locations.

A different pattern emerged in group 11, where the hierarchy was reversed: the highest N+ rate was observed in PB (72.7%), followed by PN (53.8%), and the lowest in PT (42.2%; *p* = 0.004). The effect size, expressed as Cramér’s V of 0.26 (95% CI: 0.13–0.42), corresponds to a moderate effect—smaller than in group 10, but still exceeding the threshold of clinical significance. Pairwise comparisons indicated that a statistically significant contrast occurred only between the body and tail.

In contrast, group 18 demonstrated a different, much more homogeneous profile. The N+ rate ranged from 42.3% in PN, through 53.3% in PT, to 59.1% in PB, and the global test did not reach the threshold of significance (*p* = 0.186; Table 3). Cramér’s V coefficient was only 0.14 (95% CI: 0.04–0.32), corresponding to a weak effect, bordering on negligible. None of the studied pairs reached significance.

Supplementary correlation analysis (Supplementary Materials, Section S2, Table S4) showed that the number of ELN correlated positively with the number of nodes containing metastases in all stations, with the strength of this relationship differing significantly: the highest correlation was observed in group 11 (Spearman’s ρ = 0.33; *p* < 0.001), and weaker, although still significant, relationships were observed in groups 10 (ρ = 0.20; *p* = 0.011) and 18 (ρ = 0.16; *p* = 0.037).

### 3.3. Optimal LNR Threshold

Analysis of the LNR cutoff (Table 4) revealed an optimal LNR dichotomization point of 0.08 for OS (*p* < 0.001), 0.10 for PFS-DM (*p* < 0.001), and 0.15 for PFS-LR (*p* < 0.001). The analysis performed is exploratory in nature (data-driven)—the obtained thresholds require validation in an independent cohort.

### 3.4. Kaplan–Meier Curves, Stratified by the Presence of Nodal Metastases (N+ vs. N−) at Individual Node Stations, Are Presented in Figure 4, Figure 5 and Figure 6: The Curves Were Generated Using the Entropy Balancing Method (Supplementary Materials, Section S3, Table S5, Figure S1)

**Figure 4:**
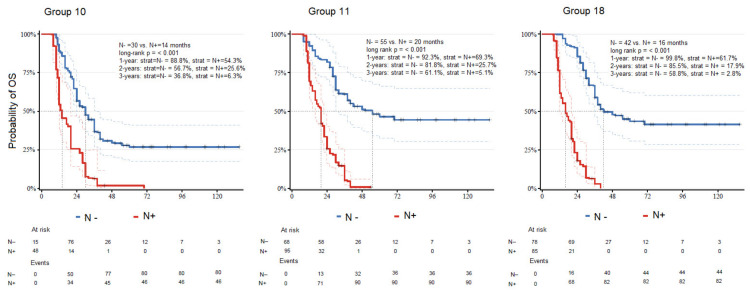
Kaplan–Meier survival curves: OS stratified by the presence of nodal metastases (N+ vs. N−) in three nodal stations—groups 10, 11, and 18. Thick, continuous lines represent the median 95% confidence interval (CI), and dashed lines illustrate the lower and upper limits of the 95% CI for each group.

The separation of OS curves (Figure 4) was most pronounced in station 18 (inferior pancreatic margin), where the median OS for N− patients reached 42 months compared to only 16 months in the N+ group, and the 1-year survival probability was nearly 100% (99.8%) in the metastasis-free subgroup, while decreasing to 61.7% among N+ patients; over the 3-year horizon, this disparity deepened to 58.8% vs. 2.8%. A similar, although slightly milder, gradient was noted in station 11 (splenic artery; median 55 vs. 20 months; 3-year OS: 61.1% vs. 5.1%) and station 10 (splenic hilum; median 30 vs. 14 months; 3-year OS: 36.8% vs. 6.3%). Absolute 3-year survival rates in the N+ groups in all three stations did not exceed several percent.

**Figure 5:**
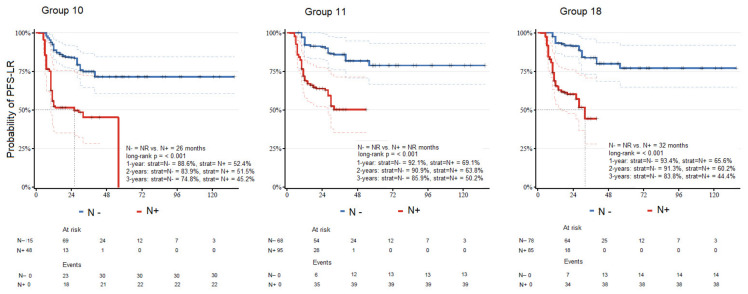
Kaplan–Meier survival curves: PFS-LR stratified by the presence of nodal metastases (N+ vs. N−) in three nodal stations—groups 10, 11, and 18. Thick, continuous lines represent the median 95% confidence interval (CI), and dashed lines illustrate the lower and upper limits of the 95% CI for each group.

**Figure 6:**
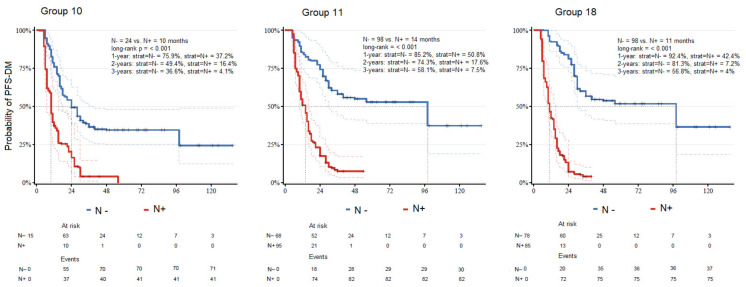
Kaplan–Meier survival curves: PFS-DM by the presence of nodal metastases (N+ vs. N−) in three nodal stations—groups 10, 11, and 18. Thick, continuous lines represent the median 95% confidence interval (CI), and dashed lines illustrate the lower and upper limits of the 95% CI for each group.

Unlike OS, the PFS-LR curves (Figure 5) showed a smaller amplitude of spread, although they retained statistical significance. In all three stations, the median PFS-LR in the N− groups was not reached (except for station 10, where the median N+ was 26 months, and station 18, where it was 32 months), and the 3-year locoregional recurrence-free probability ranged between 74.8% and 85.9% among N− patients, compared to 44.4–50.2% in the N+ subgroups.

The most distinct separation among the three analyzed endpoints was seen in the PFS-DM curves (Figure 6), confirming the crucial role of nodal metastases as a determinant of the risk of systemic dissemination. The gradient increased toward distal stations: in station 10, the median PFS-DM was 24 vs. 10 months (N− vs. N+; 3-year PFS-DM: 36.6% vs. 4.1%), in station 11–98 vs. 14 months (3-year: 58.1% vs. 7.5%), while in station 18 the disparity reached its most pronounced form: median 98 vs. 11 months, and the 2-year metastasis-free probability in the N+ group dropped to only 7.2% compared to 81.3% in the N− subgroup. The N+ curve at station 18 declined steeply over the first 12 months, then asymptotically approached zero.

### 3.5. Univariate Prognostic Factor Analysis

Univariate analysis was performed to isolate variables that demonstrated individual associations with each of the primary endpoints—OS, PFS-LR, and PFS-DM. All univariate models were estimated with entropy balancing weights (Supplementary Materials, Section S3, Table S5, Figure S1).

Demographic variables did not demonstrate significant associations with any of the three analyzed endpoints (Table 5). Age (HR per year: 1.01; *p* = 0.410 for OS), sex (HR men vs. women: 0.80; *p* = 0.322 for OS), and BMI (HR per unit: 0.98; *p* = 0.530 for OS) did not reach significance. Tumor characteristics revealed the first significant contrast: tumor size emerged as a strong, consistent factor associated with risk across all three endpoints, with hazard ratios of 1.74 (95% CI: 1.32–2.29; *p* < 0.001) for OS, 1.84 (CI: 1.45–2.34; *p* < 0.001) for PFS-DM, and 1.56 (CI: 1.01–2.43; *p* = 0.047) for PFS-LR (Table 5). Each additional centimeter of tumor size was associated with a 56–84% increased risk.

Tumor location did not reach statistical significance in any of the three univariate models. Comparison of PB vs. PN yielded HR close to unity for OS (1.05; *p* = 0.864) and PFS-LR (1.13; *p* = 0.775) and a moderately increased but non-significant HR for PFS-DM (1.40; *p* = 0.242). The PT vs. PN contrast consistently yielded higher HRs—1.55 for OS (*p* = 0.118), 1.63 for PFS-LR (*p* = 0.313), and 1.68 for PFS-DM (*p* = 0.102)—but in each case the lower bound of the 95% CI encompassed unity.

TNM staging provided the strongest predictor associated with survival in terms of effect size across the univariate comparison (Table 5). Stage with HR of 19.73 (CI: 9.11–42.71; *p* < 0.001) for OS, 13.93 (CI: 6.79–28.56) for PFS-DM, and 5.42 (CI: 2.12–13.88) for PFS-LR. These values reflect the sharp jump in risk between successive stages. The pT stage yielded HRs of 3.44 (OS; *p* < 0.001) to 3.60 (PFS-DM; *p* < 0.001), and the pN stage yielded HRs of 5.52 (PFS-LR) to 18.50 (OS), confirming the central role of the TNM classification in predicting outcomes after DP-S.

In our work, grading was a strong, consistent predictor: HR was 6.80 (CI: 4.02–11.50; *p* < 0.001) for OS, 9.44 (CI: 3.02–29.52; *p* < 0.001) for PFS-LR, and 5.40 (CI: 3.42–8.53; *p* < 0.001) for PFS-DM—meaning that each subsequent degree of loss of histological differentiation was associated with a several-fold increase in risk. PNI confirmed its prognostic value for OS (HR = 3.95; *p* < 0.001) and PFS-DM (HR = 4.14; *p* < 0.001) but did not reach significance for PFS-LR (HR = 2.05; *p* = 0.089). VI did not demonstrate a significant association with any of the three endpoints (all *p* > 0.15).

Nodal parameters constituted the largest and most clinically relevant block of the univariate analysis (Table 5). LNR showed the highest prognostic effect among continuous variables: HR = 3.47 (CI: 2.81–4.28; *p* < 0.001) for OS, 3.40 for PFS-DM, and 2.12 for PFS-LR. In the dichotomous approach (LNR > 0 vs. LNR = 0), the presence of positive lymph nodes was associated with more than an eleven-fold higher risk of death (HR = 11.52; CI: 5.60–23.70; *p* < 0.001) and almost ten-fold higher risk of distant metastases (HR = 9.91; CI: 4.75–20.68; *p* < 0.001) compared to pN0 patients. The total number of ELN did not significantly influence any of the endpoints (all *p* > 0.18), whereas the absolute number of metastatic nodes showed a consistent effect per additional node—HR = 1.39 for OS and PFS-DM (both *p* < 0.001) and 1.19 for PFS-LR (*p* < 0.001).

Each nodal station demonstrated an independent, strong prognostic association. Metastases in station 18 were associated with the highest hazard ratios: 7.78 (CI: 5.02–12.06; *p* < 0.001) for OS, 7.78 for PFS-DM, and 4.84 (CI: 2.18–10.73; *p* < 0.001) for PFS-LR. Station 11 (along splenic artery) ranked second with HR ranging from 4.35 (PFS-LR) to 5.43 (OS), and station 10 (splenic hilum): HR ranging from 3.19 (PFS-DM) to 3.94 (PFS-LR).

Neoadjuvant chemotherapy was associated with longer OS (HR = 0.60; CI: 0.38–0.94; *p* = 0.025) and PFS-DM (HR = 0.62; CI: 0.40–0.96; *p* = 0.032), but not for PFS-LR (HR = 0.60; CI: 0.30–1.20; *p* = 0.148).

### 3.6. Multivariate Analysis of Prognostic Factors

For each of the primary endpoints—OS, PFS-LR, and PFS-DM—a Cox proportional hazards model with entropy balancing weights was estimated (Supplementary Materials, Section S3, Table S5, Figure S1).

The final model for OS (Table 6) retained five independent predictors, three of which pertain to metastatic status at individual nodal stations. Metastases at station 10 (splenic hilum) were associated with a more than 3.6-fold increase in the risk of death (HR = 3.65; 95% CI: 2.34–5.70; *p* < 0.001), metastases at station 11 (along splenic artery) with a 4.4-fold increase (HR = 4.42; CI: 2.56–7.63; *p* < 0.001), and metastases at station 18 (inferior pancreatic margin) with a 3.2-fold increase (HR = 3.26; CI: 1.93–5.51; *p* < 0.001). Poorly differentiated or undifferentiated grade (G3–4) in our work was independently associated with a higher risk of death (HR 4.74; 95% CI 2.93–7.68; *p* < 0.001). Neoadjuvant chemotherapy was associated with a lower risk of death (HR = 0.57; CI: 0.39–0.84; *p* = 0.005).

The final PFS-LR model (Table 7) remained similar to the OS model regarding key predictors. Grading in our cohort remained the dominant prognostic factor (HR = 3.68; CI: 1.79–7.58; *p* < 0.001). Metastases at station 10 (HR = 4.36; CI: 2.31–8.24; *p* < 0.001), station 11 (HR = 3.44; CI: 1.40–8.42; *p* = 0.007), and station 18 (HR = 3.28; CI: 1.38–7.81; *p* = 0.007) maintained independent significance.

In the PFS-DM model (Table 8), grading again dominated (HR = 6.40; CI: 3.77–10.87; *p* < 0.001) and all three nodal stations maintained independent significance: station 10 (HR = 4.39; CI: 2.08–9.25; *p* < 0.001), station 18 (HR = 2.94; CI: 1.68–5.15; *p* < 0.001), and station 11 (HR = 3.50; CI: 1.92–6.39; *p* < 0.001). Neoadjuvant chemotherapy was associated with a lower risk of distant metastases (HR = 0.62; CI: 0.41–0.94; *p* = 0.024).

Tumor location in this model was not statistically significant: the contrast between PB and PN yielded an HR of 1.47 (CI: 0.87–2.48; *p* = 0.147), and between PT and PN yielded an HR of 0.76 (CI: 0.33–1.75; *p* = 0.516).

Nodal-station and neoadjuvant effects satisfied the proportional-hazards assumption in all models (all *p* > 0.10). Grading showed a non-proportional pattern for OS (*p* = 0.004) and PFS-DM (*p* = 0.008); its hazard ratio is therefore reported as a time-averaged effect. A grade-by-time interaction confirmed attenuation over time without affecting the nodal-station estimates (Supplementary Materials, Section S5, Table S7).

After bootstrap optimism correction, the models retained strong discrimination (corrected Harrell’s C 0.80–0.83) with negligible optimism (≤0.008) (Supplementary Materials, Section S4, Table S6), and the optimism-corrected calibration slope remained close to unity (0.92–0.95) (Supplementary Materials, Section S6, Table S8), indicating well-calibrated and minimally over-fitted models.

## 4. Discussion

### 4.1. The Significance of Lymph Node Metastases in PDAC

The lymph node status in patients with PDAC is an important prognostic factor for overall survival and disease-free survival in patients who have undergone surgery [11], and lymphadenectomy itself is essential for the proper assessment of the disease stage (pN feature) [8]. In the currently valid eighth edition of the American Joint Committee on Cancer (AJCC) classification, lymph nodes have additionally been categorized according to the number of positive nodes: category pN0—no lymph node metastases, category pN1—metastases to 1–3 regional lymph nodes, and category pN2—metastases in 4 or more regional lymph nodes [12].

According to our findings, nodal metastases have a significant impact on the prognosis after DP-S procedures. The absolute 3-year survival rates in the N+ groups across all three nodal stations did not exceed a few percent—this observation confirms the deeply unfavorable effect of nodal metastases on long-term prognosis, regardless of the specific anatomical location of the affected nodes. An interesting observation regarding prognosis is related to PFS-LR, where the Kaplan–Meier curves reach a plateau after the first 12–18 months of observation—both in the N− and N+ subgroups—which reflects the fact that local recurrences, if they are to occur, appear within a relatively narrow time frame after surgery, and patients who survive this critical period without recurrence maintain a stable probability of further recurrence-free survival.

In the PFS-DM analysis, the N+ curve for station 18 dropped sharply during the first 12 months and then asymptotically approached zero—a characteristic profile for cancer with a high tendency for early hematogenous spread [13]. Therefore, the results of our study highlight the role of lymph node metastases as a determinant of the risk of distant dissemination—in the case of PFS-DM, the separation of the N+/N− curves was the deepest and earliest. Involvement of regional lymph nodes is thus also a marker of systemic disease advancement, not just the extent of the locoregional process. We believe that it is precisely patients with histopathologically confirmed lymph node metastases who should receive particularly thorough oncological monitoring after DP-S surgery.

### 4.2. Number of Examined Lymph Nodes (ELN)

In our study, the median number of examined lymph nodes (ELN) did not differ significantly between groups (*p* = 0.085) and was 23 nodes in the PN group, 25 in the PB group, and 25 in the PT group. On the other hand, the number of metastatic lymph nodes (N+) in the respective nodal groups was statistically significant (*p* = 0.013) and was 1 in the PN group, 3 in the PB group, and 4 in the PT group. An additional correlation analysis (Supplementary Materials, Section S2, Table S4) showed that the extent of lymphadenectomy—measured by the number of ELN—positively correlated with the number of nodes containing metastases in all stations, with the strength of this association varying significantly: the highest correlation was observed in group 11 (Spearman ρ = 0.33; *p* < 0.001), while weaker, though still significant, correlations were observed in groups 10 (ρ = 0.20; *p* = 0.011) and 18 (ρ = 0.16; *p* = 0.037). This result suggests that at station 11, the expansion of the range of examined nodes brings the proportionally greatest diagnostic gain in detecting metastatic disease, which may translate into more accurate pathological pN staging and more precise prognosis.

According to ISGPS guidelines, the numerical range for lymphadenectomy should be ≥15 lymph nodes. A recent study by Malleo et al. [13] indicated that the minimum ELN ensuring adequate assessment of disease staging during distal PDAC resection should be 20. It has been shown that such a threshold has a strong prognostic impact, mainly in patients without lymph node involvement, with a threefold increase in disease-related mortality in patients with ELN < 20. Similar conclusions were drawn by the authors of the DIPLOMA study [14]—according to their findings as well, to achieve an accurate determination of the N stage and low lymph node rates, it is important to examine at least 20 lymph nodes. In the study by Ashfaq A et al. [15], in the subgroup without lymph node involvement, the median and 5-year overall survival in patients with ELN ≤ 10 were significantly worse than in patients with ELN > 10 (16 vs. 20 months and 13 vs. 19%, respectively, *p* < 0.011). The authors believe that in patients with PDAC undergoing distal pancreatectomy, at least 11 lymph nodes should be examined to avoid underestimating the stage of the disease. In our entire cohort, the median ELN was 24, which significantly exceeds values reported in similar studies [8,13,15]. These results underscore the thoroughness and care we took in performing lymphadenectomies during DP-S procedures. However, it should be noted that these results do not resolve the uncertainty regarding optimal ELN cutoff values. The issue of ELN requires further multicenter studies and perhaps, in the future, changes to the current guidelines.

### 4.3. Lymph Node Ratio (LNR)

Numerous basic studies show that LNR is significantly more effective compared to lymph node involvement alone (N0/N+) and the ELN in prognostic stratification [15,16,17,18]. In our study, an exploratory analysis of the LNR cutoff (Table 4), conducted using the method of maximally selected standardized statistics, revealed the optimal LNR dichotomization point at 0.08 for overall survival (OS; statistic = 9.46; *p* < 0.001), 0.10 for distant metastasis-free survival (PFS-DM; statistic = 9.28; *p* < 0.001), and 0.15 for local recurrence-free survival (PFS-LR; statistic = 5.63; *p* < 0.001). In our cohort, even with a relatively small nodal metastatic burden—at the level of 8–15% positive nodes—there is a significant change in the risk profile. However, it should be clearly emphasized that the cutoffs (0.08, 0.10, 0.15) are exploratory and may be overfit to this single cohort, limiting immediate clinical application.

Similar results were obtained in the study by Ashfaq A et al. [15], in which patients with LNR ≤ 0.1 had better 5-year overall survival compared to those with LNR > 0.1 (17 vs. 6%, *p* = 0.002). These are significant findings in the context of the previously most commonly used LNR threshold of 0.2 for prognostic stratification in PDAC [16,17].

### 4.4. Pattern of Metastasis to Regional Lymph Nodes in the Case of PDAC of the Distal Part of the Pancreas

In this study, the pattern of metastasis to specific lymph node stations was found to be strictly dependent on tumor location. Involvement of group 10 (*p* < 0.001) was characteristic for tumors located in the PT (84.4%; 95% CI: 70–93%). Pairwise analyses (Supplementary Materials, Section S1, Table S2) confirmed that the source of this effect was exclusively the pairs involving the tail: both PN–PT contrasts (pFDR ~ 10^−16^) and PB–PT (pFDR ~ 10^−15^) reached *p*-values of extremely low orders of magnitude, while the PN–PB comparison showed no difference (*p* = 0.509; Supplementary Materials, Section S1, Table S3). This observation corresponds with the anatomy of lymphatic drainage of the pancreatic tail, which runs directly toward the splenic hilum [9]—and provides strong support for the thesis that tumors located in this segment exhibit a highly specific metastatic tropism toward the splenic station. Moreover, such extreme variation in N+ rates (more than a fourteen-fold difference between the tail and the isthmus) increases the value of this result in the context of potential planning of the extent of lymphadenectomy. Similar conclusions were reported by Matsui Y et al. [19]—patients with PT lymph nodes in the splenic hilum had the highest rate of metastases (38.1%).

The authors of the study by Hirashita et al. [20] reached different conclusions—in patients with PDAC in the pancreatic tail (PT), a high frequency of metastases to the lymph nodes along the splenic artery (39%) was observed. In turn, in the study by Tanaka et al. [21], in patients with a tumor in the PT, metastases occurred in the lymph nodes of groups 10, 11, and 18. In the multicenter study by Tanaka et al. [22], which was a continuation of previous studies [16,17,18,19,20,21], no metastases to the lymph nodes of group 10 were found in patients with PB tumors.

Involvement of group 11 (*p* = 0.004) in our study was characteristic of tumors located in the PB (72.7%; 95% CI: 60–83%). Such a formed gradient reflects the topographical proximity of body tumors to the course of the splenic artery and the accompanying lymphatic collectors—the dominant pathway of lymphatic drainage from the central part of the organ [9]. From a surgical perspective, this finding emphasizes the necessity of particularly meticulous lymphadenectomy along the splenic artery in patients with PB tumors, where almost three-quarters of the nodes in this station are found to contain metastases. Interestingly, in the context of the results we obtained, in the study by Tanaka [21], patients with a tumor in PN or PB also had metastases to group 11 lymph nodes, but only to its proximal part (11p), without involvement of the distal part (11d). In our study, we did not subdivide group 11 into 11p and 11d, which precluded direct comparison with studies that used such subdivision.

Lymphatic drainage of group 18 in our study constitutes a “common zone”, engaged regardless of PDAC location (*p* = 0.185). This lack of significant differences in group 18 is clinically informative and no less important than the pronounced contrasts in stations 10 and 11. Lymphatic drainage of the lower margin of the pancreas functions as a “common zone” for the entire distal segment of the organ, involved in the metastatic process with comparable probability, whether the primary focus resides in PN, PB, or PT. The clinical consequence of this phenomenon is clear: precise lymphadenectomy of group 18 nodes retains its oncological rationale in every case of distal resection, regardless of PDAC location.

### 4.5. Factors Affecting the Prognosis of Patients After DP-S Due to PDAC

In our study, the degree of histological differentiation (grading) constitutes the strongest independent factor, which is associated with survival at all three endpoints. Metastatic status in individual lymph node stations—Gr. 10, 11, and 18—retains an independent, additive prognostic value, which may serve as a strong argument for reporting N+ status per station, rather than solely the overall pN category, in the pathological assessment of material after distal pancreatectomy. Tumor location, despite a trend toward higher risk for PT, was not independently associated with survival at any endpoint; its effect appears to be indirect, mediated through an anatomically specific pattern of metastasis to regional lymph nodes. It is an important observation from our study that tumor location in the distal part of the pancreas exerts its impact primarily through anatomic drainage patterns and associated nodal involvement, rather than serving as an independent prognostic factor.

The significance of lymph node metastases is, in numerous publications, a factor affecting prognosis after DP-S procedures due to PDAC. In a multicenter study by Tanaka et al. [22], in a multivariate analysis, metastases in lymph nodes along the splenic artery were identified as an independent predictive factor of poor overall survival in patients with body and tail pancreatic cancer. Korrel et al. [23] found that resection of Gerota’s fascia (HR 0.74; *p* = 0.019), R0 resection (HR 0.70; *p* = 0.006), reduced LNR (HR 0.28; *p* < 0.001), and adjuvant chemotherapy (HR 0.67; *p* = 0.003) were associated with improved overall survival. In the study by Ishida [24], which analyzed data from 110 patients with invasive pancreatic cancer who underwent distal pancreatectomy, multivariate survival analysis showed that tumor size > 4 cm (HR: 2.23, *p* = 0.012), metastases to lymph nodes other than peripancreatic ones (HR: 3.02, *p* = 0.015), and inability to undergo adjuvant chemotherapy (HR: 2.81, *p* = 0.0018) were associated with poor prognosis.

However, there are reports of no effect of nodal factors on prognosis. In the study by Hirashita T et al. [20], in patients with PDAC in PT, a multivariate analysis showed that tumor size was the only independent factor associated with recurrence-free survival (HR = 2.01, 95% CI = 1.33–3.05, *p* = 0.001). In our study, the size of PDAC was significantly larger in PT—median 4.0 cm (IQR: 3.0–4.0), exceeding both PN (median 2.5 cm) and PB (median 3.0 cm)—in the univariate analysis, tumor size reached statistical significance at all endpoints, but in the multivariate analysis, it did not meet the significance criteria at any endpoint.

It is possible that the development of modern diagnostic techniques, including multimodal imaging by combining nuclear medicine tracers with other non-radioactive molecular probes and/or biomedical imaging techniques, will contribute to more accurate and consistent diagnosis and treatment of PDAC [25].

### 4.6. Impact of Neoadjuvant Treatment

Neoadjuvant treatment, for example, neoadjuvant chemotherapy, especially used in borderline PDAC (BR-PDAC) or locally advanced PDAC (LA-PDAC), can shrink the tumor, potentially making it resectable (downstaging), which may translate into better survival outcomes [26]. The benefits of using neoadjuvant treatment may also concern the lymph nodes. In the study by Roland et al. [27], patients who underwent neoadjuvant therapy and then surgery had better overall survival and time to local recurrence compared to the surgery-first approach. These patients also had a lower likelihood of lymph node metastasis (*p* = 0.001). In the study by Portundo et al. [28], they analyzed patients who were clinically positive in lymph nodes (cN+) before treatment. They reported that neoadjuvant therapy achieved pathological reduction in lymph node involvement (to a negative nodal status) in about 38% of such patients. Importantly, patients who achieved a negative nodal status had significantly better survival compared to those who remained node-positive (HR for death about 0.61).

In turn, Phase III of the PREOPANC study [29] compared gemcitabine-based neoadjuvant chemoradiotherapy followed by surgical treatment with immediate surgery in resectable/borderline PDAC. Although patients receiving preoperative chemoradiotherapy showed, among other things, a lower rate of lymph node metastases compared to the group undergoing immediate surgery, the difference in overall survival was not significant.

In our study, the use of neoadjuvant chemotherapy was associated with a lower risk of death and distant metastasis in this retrospective cohort, both in univariate and multivariate analyses. However, at our center, NAT is administered only for BR-PDAC and LA-PDAC, with the FOLFIRINOX regimen. For all resectable tumors, upfront surgery is performed. This non-random selection of groups makes the causal effect between the use of neoadjuvant chemotherapy and improved survival highly susceptible to indication bias and unmeasured confounding factors—these results are hypothesis-generating and should not be interpreted as proof of a protective effect of neoadjuvant therapy.

Currently, there is no convincing evidence for the widespread use of NAT in patients with resectable PDAC [30], so despite our promising results regarding improved prognosis for PDAC patients after NAT, further multicenter studies are necessary.

### 4.7. Study Limitations

Our study had some limitations. It was a retrospective single-center cohort study with potential selection bias.

Some patients were excluded from the study (*N* = 24), including patients with non-R0 resection (*N* = 14), which could have affected the outcome and reduced the strength of conclusions from the statistical analysis.

In addition, in our study, we did not differentiate group 11 into 11p and 11d, which limits the possibility of comparing results with studies in which this division was used.

Our study analyzes only the DP-S procedure with standard lymphadenectomy, so we cannot compare our results with those of studies based on other surgical approaches and extended lymphadenectomy.

Although FDR control was applied within families, the exploratory analyses involved many comparisons, and the associations they generated should be regarded as hypothesis-generating and confirmed in independent cohorts.

Patients were operated on by eight different consultant surgeons during the study period (13 years), which may contribute to some heterogeneity in the surgical approach. Additionally, guidelines regarding standards for histopathological examination also changed over time. Furthermore, there is limited generalizability of station-specific patterns to centers with different surgical practices. Due to the lack of widely used central guidelines for radiological and pathological examination, comparison with other works on this subject may be difficult.

## 5. Conclusions

The location of PDAC in the distal pancreas influences metastasis to specific lymph nodes: group 10 involvement (*p* < 0.001) is characteristic for tumor location in the PT (84.4%; 95% CI: 70–93%), and group 11 involvement (*p* = 0.004) for tumors located in the PB (72.7%; 95% CI: 60–83%). Lymphatic drainage in group 18 constitutes a common zone, involved regardless of PDAC location (*p* = 0.185). Metastasis to regional lymph nodes is a predictor of survival after DP-S procedures, primarily determining the risk of distant metastases. In our retrospective cohort, neoadjuvant chemotherapy is associated with a lower risk of death and distant metastases due to PDAC after DP-S procedures.

## Figures and Tables

**Table 1:** Clinicopathological characteristics of the study population by tumor location. Statistically significant values (*p* < 0.05) are bolded; *p*-values in Table 1 are descriptive and did not inform model specification. PN = pancreatic neck; PB = pancreatic body; PT = pancreatic tail; BMI = body mass index; ASA = American Society of Anesthesiologists (anesthetic risk classification); ELN = examined lymph nodes—number of all lymph nodes examined during histopathological examination; LNR = lymph node ratio—the ratio of the number of lymph nodes with PDAC metastases to the total number of examined lymph nodes; PNI = perineural invasion; VI = vascular invasion; OS = overall survival; CI = confidence interval.

Variable	Total (*N* = 163)	95% CI	PN (*n* = 52)	95% CI	PB (*n* = 66)	95% CI	PT (*n* = 45)	95% CI	*p*
**Demographic data**									
Age [years]	Median: 67.0 (60.0–70.0)	63–65	Median: 66.5 (59.5–70.0)	60–66	Median: 66.0 (60.0–69.0)	61–65	Median: 67.0 (62.0–70.0)	64–68	0.229
Sex, *n* (%)									0.960
Women	83 (50.9%)	43–59%	27 (51.9%)	38–66%	34 (51.5%)	39–64%	22 (48.9%)	34–64%	
Men	80 (49.1%)	41–57%	25 (48.1%)	34–62%	32 (48.5%)	36–61%	23 (51.1%)	36–66%	
BMI [kg/m^2^]	Median: 24.1 (22.8–26.3)	24–25	Median: 24.7 (23.1–28.3)	24–26	Median: 24.1 (22.8–25.7)	23–25	Median: 23.9 (22.6–26.1)	23–25	0.121
Smoking, *n* (%)									0.128
No	79 (48.5%)	41–56%	31 (59.6%)	45–73%	27 (40.9%)	29–54%	21 (46.7%)	32–62%	
Yes	84 (51.5%)	44–59%	21 (40.4%)	27–55%	39 (59.1%)	46–71%	24 (53.3%)	38–68%	
**Tumor features**									
Size [cm]	Median: 3.0 (2.0–3.5)	2.8–3.1	Median: 2.5 (2.0–3.0)	2.4–2.9	Median: 3.0 (2.0–3.0)	2.5–2.9	Median: 4.0 (3.0–4.0)	3.4–4.1	**<0.001**
Resectability, *n* (%)									0.814
Resectable	88 (54.0%)	46–62%	30 (57.7%)	43–71%	34 (51.5%)	39–64%	24 (53.3%)	38–68%	
Borderline	75 (46.0%)	38–54%	22 (42.3%)	29–57%	32 (48.5%)	36–61%	21 (46.7%)	32–62%	
Grading, *n* (%)									0.992
G1	28 (17.2%)	12–24%	9 (17.3%)	8.7–31%	10 (15.2%)	7.9–27%	9 (20.0%)	10–35%	
G2	102 (62.6%)	55–70%	33 (63.5%)	49–76%	42 (63.6%)	51–75%	27 (60.0%)	44–74%	
G3	32 (19.6%)	14–27%	10 (19.2%)	10–33%	13 (19.7%)	11–32%	9 (20.0%)	10–35%	
G4	1 (0.6%)	0.03–3.9%	0 (0.0%)	0.00–8.6%	1 (1.5%)	0.08–9.3%	0 (0.0%)	0.00–9.8%	
Staging, *n* (%)									**0.035**
I	47 (28.8%)	22–37%	22 (42.3%)	29–57%	19 (28.8%)	19–41%	6 (13.3%)	5.5–27%	
II	48 (29.4%)	23–37%	13 (25.0%)	14–39%	20 (30.3%)	20–43%	15 (33.3%)	20–49%	
III	68 (41.7%)	34–50%	17 (32.7%)	21–47%	27 (40.9%)	29–54%	24 (53.3%)	38–68%	
pT, *n* (%)									**<0.001**
pT1	55 (33.7%)	27–42%	24 (46.2%)	32–60%	26 (39.4%)	28–52%	5 (11.1%)	4.2–25%	
pT2	87 (53.4%)	45–61%	20 (38.5%)	26–53%	38 (57.6%)	45–69%	29 (64.4%)	49–78%	
pT3	21 (12.9%)	8.3–19%	8 (15.4%)	7.3–29%	2 (3.0%)	0.53–11%	11 (24.4%)	13–40%	
pN, *n* (%)									**0.025**
pN0	50 (30.7%)	24–38%	24 (46.2%)	32–60%	19 (28.8%)	19–41%	7 (15.6%)	7.0–30%	
pN1	45 (27.6%)	21–35%	11 (21.2%)	12–35%	20 (30.3%)	20–43%	14 (31.1%)	19–47%	
pN2	68 (41.7%)	34–50%	17 (32.7%)	21–47%	27 (40.9%)	29–54%	24 (53.3%)	38–68%	
Total examined lymph nodes (ELN)	Median: 24.0 (20.0–28.0)	24–25	Median: 23.0 (20.0–25.0)	22–24	Median: 25.0 (21.0–30.0)	24–26	Median: 25.0 (21.0–29.0)	23–26	0.085
Total metastatic lymph nodes	Median: 3.0 (0.0–7.0)	3.6–5.1	Median: 1.0 (0.0–5.5)	1.8–4.0	Median: 3.0 (0.0–8.0)	3.6–6.2	Median: 4.0 (1.0–7.0)	3.7–6.5	**0.013**
LNR	Median: 0.1 (0.0–0.3)	0.14–0.20	Median: 0.0 (0.0–0.2)	0.08–0.17	Median: 0.1 (0.0–0.3)	0.14–0.24	Median: 0.2 (0.1–0.4)	0.15–0.25	**0.022**
LNR (dichotomous), *n* (%)									**0.004**
LNR = 0	49 (30.1%)	23–38%	24 (46.2%)	32–60%	18 (27.3%)	17–40%	7 (15.6%)	7.0–30%	
LNR > 0	114 (69.9%)	62–77%	28 (53.8%)	40–68%	48 (72.7%)	60–83%	38 (84.4%)	70–93%	
PNI, *n* (%)									0.075
No	44 (27.0%)	20–35%	19 (36.5%)	24–51%	12 (18.2%)	10–30%	13 (28.9%)	17–45%	
Yes	119 (73.0%)	65–80%	33 (63.5%)	49–76%	54 (81.8%)	70–90%	32 (71.1%)	55–83%	
VI, *n* (%)									0.446
No	61 (37.4%)	30–45%	23 (44.2%)	31–59%	22 (33.3%)	23–46%	16 (35.6%)	22–51%	
Yes	102 (62.6%)	55–70%	29 (55.8%)	41–69%	44 (66.7%)	54–77%	29 (64.4%)	49–78%	
**Treatment**									
Neoadjuvant chemotherapy, *n* (%)									0.541
No	81 (49.7%)	42–58%	29 (55.8%)	41–69%	30 (45.5%)	33–58%	22 (48.9%)	34–64%	
Yes	82 (50.3%)	42–58%	23 (44.2%)	31–59%	36 (54.5%)	42–67%	23 (51.1%)	36–66%	
ASA, *n* (%)									0.653
I	52 (31.9%)	25–40%	17 (32.7%)	21–47%	18 (27.3%)	17–40%	17 (37.8%)	24–53%	
II	101 (62.0%)	54–69%	33 (63.5%)	49–76%	42 (63.6%)	51–75%	26 (57.8%)	42–72%	
III	10 (6.1%)	3.1–11%	2 (3.8%)	0.67–14%	6 (9.1%)	3.7–19%	2 (4.4%)	0.77–16%	
Complications according to Clavien–Dindo, *n* (%)									0.479
0—no complications	74 (45.4%)	38–53%	26 (50.0%)	37–63%	32 (48.5%)	36–61%	16 (35.6%)	22–51%	
I	25 (15.3%)	10–22%	8 (15.4%)	7.3–29%	12 (18.2%)	10–30%	5 (11.1%)	4.2–25%	
II	20 (12.3%)	7.8–19%	6 (11.5%)	4.8–24%	5 (7.6%)	2.8–18%	9 (20.0%)	10–35%	
III	34 (20.9%)	15–28%	8 (15.4%)	7.3–29%	14 (21.2%)	12–33%	12 (26.7%)	15–42%	
IV	10 (6.1%)	3.1–11%	4 (7.7%)	2.5–19%	3 (4.5%)	1.2–14%	3 (6.7%)	1.7–19%	
CA 19-9 [U/mL]	Median: 88.0 (49.0–145.0)	95–119	Median: 89.0 (50.5–162.0)	91–142	Median: 89.0 (52.0–136.0)	89–127	Median: 83.0 (39.0–129.0)	73–116	0.417
**Endpoints**									
Local recurrence, *n* (%)									0.597
No	111 (68.1%)	60–75%	36 (69.2%)	55–81%	47 (71.2%)	59–81%	28 (62.2%)	47–76%	
Yes	52 (31.9%)	25–40%	16 (30.8%)	19–45%	19 (28.8%)	19–41%	17 (37.8%)	24–53%	
Distant metastases after operation, *n* (%)									**0.041**
No	51 (31.3%)	24–39%	23 (44.2%)	31–59%	15 (22.7%)	14–35%	13 (28.9%)	17–45%	
Yes	112 (68.7%)	61–76%	29 (55.8%)	41–69%	51 (77.3%)	65–86%	32 (71.1%)	55–83%	
Death, *n* (%)									0.125
No	37 (22.7%)	17–30%	16 (30.8%)	19–45%	15 (22.7%)	14–35%	6 (13.3%)	5.5–27%	
Yes	126 (77.3%)	70–83%	36 (69.2%)	55–81%	51 (77.3%)	65–86%	39 (86.7%)	73–94%	
Overall survival, OS [months]	Median: 24.0 (14.0–38.0)	28–35	Median: 29.0 (18.5–41.0)	29–45	Median: 24.0 (14.0–39.0)	25–37	Median: 20.0 (12.0–32.0)	21–31	0.102

**Table 2:** Distribution of lymph node metastases by anatomical station and tumor location. Statistically significant values (*p* < 0.05) are bolded. PN = pancreatic neck; PB = pancreatic body; PT = pancreatic tail; ELN = examined lymph nodes; LNR = lymph node ratio—the ratio of the number of lymph nodes with PDAC metastases to the total number of examined lymph nodes; N+ = ≥1 lymph node with metastases; N− = no metastases in a given station; CI = confidence interval.

Variable	Total (*N* = 163)	95% CI	PN (*n* = 52)	95% CI	PB (*n* = 66)	95% CI	PT (*n* = 45)	95% CI	*p*
Group 10									
Examined lymph nodes (ELN)	Median: 10.0 (9.0–11.0)	9.7–10	Median: 10.0 (8.0–10.0)	9.10–10.00	Median: 10.0 (9.0–10.0)	8.90–10.00	Median: 10.0 (10.0–14.0)	10–12	**0.010**
Metastatic lymph nodes	Median: 0.0 (0.0–1.0)	0.76–1.5	Median: 0.0 (0.0–0.0)	0.02–0.25	Median: 0.0 (0.0–0.0)	0.00–0.79	Median: 2.0 (1.0–5.0)	2.4–4.2	**<0.001**
LNR	Median: 0.0 (0.0–0.1)	0.07–0.13	Median: 0.0 (0.0–0.0)	0.00–0.03	Median: 0.0 (0.0–0.0)	0.00–0.06	Median: 0.3 (0.1–0.5)	0.22–0.38	**<0.001**
Metastatic status, *n* (%)									**<0.001**
N− (without metastases)	115 (70.6%)	63–77%	49 (94.2%)	83–98%	59 (89.4%)	79–95%	7 (15.6%)	7–30%	
N+ (with metastases)	48 (29.4%)	23–37%	3 (5.8%)	1.5–17%	7 (10.6%)	4.7–21%	38 (84.4%)	70–93%	
**Group 11**									
Examined lymph nodes (ELN)	Median: 6.0 (5.0–8.0)	6.4–7.1	Median: 6.0 (5.5–7.5)	6.1–7.1	Median: 7.0 (6.0–10.0)	7.3–8.3	Median: 5.0 (4.0–7.0)	4.8–6.0	**<0.001**
Metastatic lymph nodes	Median: 1.0 (0.0–3.0)	1.4–2.0	Median: 1.0 (0.0–3.0)	0.94–2.1	Median: 2.0 (0.0–5.0)	1.9–3.1	Median: 0.0 (0.0–1.0)	0.40–1.0	**<0.001**
LNR	Median: 0.1 (0.0–0.4)	0.20–0.29	Median: 0.1 (0.0–0.4)	0.16–0.33	Median: 0.2 (0.0–0.6)	0.25–0.41	Median: 0.0 (0.0–0.2)	0.07–0.19	**0.002**
Metastatic status, *n* (%)									**0.004**
N− (without metastases)	68 (41.7%)	34–50%	24 (46.2%)	32–60%	18 (27.3%)	17–40%	26 (57.8%)	42–72%	
N+ (with metastases)	95 (58.3%)	50–66%	28 (53.8%)	40–68%	48 (72.7%)	60–83%	19 (42.2%)	28–58%	
**Group 18**									
Examined lymph nodes (ELN)	Median: 7.0 (6.0–10.0)	7.1–7.8	Median: 6.0 (5.0–8.0)	6.1–7.3	Median: 8.0 (6.0–10.0)	7.2–8.3	Median: 8.0 (6.0–10.0)	7.1–8.4	**0.025**
Metastatic lymph nodes	Median: 1.0 (0.0–2.0)	1.2–1.8	Median: 0.0 (0.0–2.0)	0.67–1.7	Median: 1.0 (0.0–4.0)	1.4–2.6	Median: 1.0 (0.0–2.0)	0.67–1.4	0.106
LNR	Median: 0.1 (0.0–0.3)	0.16–0.23	Median: 0.0 (0.0–0.3)	0.11–0.25	Median: 0.1 (0.0–0.5)	0.18–0.32	Median: 0.1 (0.0–0.2)	0.09–0.18	0.105
Metastatic status, *n* (%)									0.185
N− (without metastases)	78 (47.9%)	40–56%	30 (57.7%)	43–71%	27 (40.9%)	29–54%	21 (46.7%)	32–62%	
N+ (with metastases)	85 (52.1%)	44–60%	22 (42.3%)	29–57%	39 (59.1%)	46–71%	24 (53.3%)	38–68%	

**Table 3:** Frequency of nodal metastases (N+) by anatomical station and tumor location. Statistically significant values (*p* < 0.05) are bolded. N+ = ≥1 metastasis in a station. PN = pancreatic neck; PB = pancreatic body; PT = pancreatic tail; CI = confidence interval.

Lymph Nodes Station	PN *n* (%)	PB *n* (%)	PT *n* (%)	*p*	Cramér’s V (95% CI)
Group 10	3 (5.8%)	7 (10.6%)	38 (84.4%)	**<0.001**	0.75 (0.64–0.86)
Group 11	28 (53.8%)	48 (72.7%)	19 (42.2%)	**0.004**	0.26 (0.13–0.42)
Group 18	22 (42.3%)	39 (59.1%)	24 (53.3%)	0.186	0.14 (0.04–0.32)

**Table 4:** Exploratory determination of the optimal LNR cutoff using the maximum standardized statistics per endpoint method—the results of this analysis are hypothesis-generating and require external validation. Statistically significant values (*p* < 0.05) are bolded. OS = overall survival; PFS-LR = progression-free survival local recurrence; PFS-DM = progression-free survival distant metastases; LNR = lymph node ratio—the ratio of the number of lymph nodes with PDAC metastases to the total number of examined lymph nodes.

Endpoint	Optimal LNR	Statistics	*p*	Clinical Interpretation
OS	0.08	9.46	**<0.001**	≥8% metastatic nodes
PFS-LR	0.15	5.63	**<0.001**	≥15% metastatic nodes
PFS-DM	0.10	9.28	**<0.001**	≥10% metastatic nodes

**Table 5:** Univariate analysis of prognostic factors using the Cox proportional hazards model with entropy balancing weights—hazard ratios (HR) with 95% CI. ^a^ LNR × 10 − HR reflects an increase in risk for every 0.1 unit increase in LNR. PN = pancreatic neck; PB = pancreatic body; PT = pancreatic tail; OS = overall survival; PFS-LR = progression-free survival local recurrence; PFS-DM = progression-free survival distant metastases; HR = hazard ratio; CI = confidence interval; PNI = perineural invasion; VI = vascular invasion; LNR = lymph node ratio; ELN = examined lymph nodes. Statistically significant values (*p* < 0.05) are bolded.

	OS		PFS-LR		PFS-DM	
Variable	HR (95% CI)	*p*	HR (95% CI)	*p*	HR (95% CI)	*p*
**Demographic data**						
Age [years]	1.01 (0.99–1.04)	0.410	1.02 (0.98–1.07)	0.303	1.01 (0.98–1.04)	0.526
Sex (men vs. women)	0.80 (0.51–1.25)	0.322	0.55 (0.27–1.11)	0.096	0.76 (0.49–1.20)	0.238
BMI [kg/m^2^]	0.98 (0.90–1.05)	0.530	1.03 (0.90–1.18)	0.639	0.99 (0.91–1.07)	0.723
**Tumor features**						
Tumor size [cm]	1.74 (1.32–2.29)	**<0.001**	1.56 (1.01–2.43)	**0.047**	1.84 (1.45–2.34)	**<0.001**
Location: PB vs. PN	1.05 (0.60–1.82)	0.864	1.13 (0.48–2.64)	0.775	1.40 (0.80–2.45)	0.242
Location: PT vs. PN	1.55 (0.89–2.68)	0.118	1.63 (0.63–4.24)	0.313	1.68 (0.90–3.12)	0.102
**TNM**						
Staging	19.73 (9.11–42.71)	**<0.001**	5.42 (2.12–13.88)	**<0.001**	13.93 (6.79–28.56)	**<0.001**
pT	3.44 (2.28–5.18)	**<0.001**	2.63 (1.09–6.32)	**0.031**	3.60 (2.33–5.56)	**<0.001**
pN	18.50 (8.76–39.07)	**<0.001**	5.52 (2.16–14.16)	**<0.001**	13.33 (6.68–26.62)	**<0.001**
**Histopathological examination results**						
Grading (G3–4 vs. G1–2)	6.80 (4.02–11.50)	**<0.001**	9.44 (3.02–29.52)	**<0.001**	5.40 (3.42–8.53)	**<0.001**
PNI (yes vs. no)	3.95 (2.18–7.16)	**<0.001**	2.05 (0.90–4.71)	0.089	4.14 (2.30–7.45)	**<0.001**
VI (yes vs. no)	1.34 (0.86–2.08)	0.201	1.13 (0.53–2.40)	0.751	1.37 (0.89–2.11)	0.152
**Node parameters**						
LNR (HR per 0.1 increase) ^a^	3.47 (2.81–4.28)	**<0.001**	2.12 (1.56–2.88)	**<0.001**	3.40 (2.81–4.12)	**<0.001**
LNR > 0 vs. LNR = 0	11.52 (5.60–23.70)	**<0.001**	4.38 (1.53–12.55)	**0.006**	9.91 (4.75–20.68)	**<0.001**
ELN (total)	0.98 (0.93–1.02)	0.286	0.99 (0.92–1.06)	0.818	0.97 (0.93–1.02)	0.188
Metastatic lymph nodes (total)	1.39 (1.29–1.49)	**<0.001**	1.19 (1.12–1.27)	**<0.001**	1.39 (1.30–1.47)	**<0.001**
Gr. 10: N+ vs. N−	3.32 (2.15–5.13)	**<0.001**	3.94 (1.93–8.02)	**<0.001**	3.19 (2.06–4.94)	**<0.001**
Gr. 11: N+ vs. N−	5.43 (3.11–9.47)	**<0.001**	4.35 (1.89–10.01)	**<0.001**	4.74 (2.70–8.31)	**<0.001**
Gr. 18: N+ vs. N−	7.78 (5.02–12.06)	**<0.001**	4.84 (2.18–10.73)	**<0.001**	7.78 (4.88–12.41)	**<0.001**
**Treatment**						
ChT Neo (yes vs. no)	0.60 (0.38–0.94)	**0.025**	0.60 (0.30–1.20)	0.148	0.62 (0.40–0.96)	**0.032**

**Table 6:** Multivariate Cox analysis—OS. Statistically significant values (*p* < 0.05) are bolded. HR = hazard ratio with 95% CI, models with entropy balancing weights.

Variable	HR (95% CI)	*p*
Grading (G3–4 vs. G1–2)	4.74 (2.93–7.68)	**<0.001**
Neoadjuvant chemotherapy (yes vs. no)	0.57 (0.39–0.84)	**0.005**
Gr. 10: N+ vs. N−	3.65 (2.34–5.70)	**<0.001**
Gr. 11: N+ vs. N−	4.42 (2.56–7.63)	**<0.001**
Gr. 18: N+ vs. N−	3.26 (1.93–5.51)	**<0.001**

**Table 7:** Cox multivariate analysis—PFS-LR. Statistically significant values (*p* < 0.05) are bolded. HR = hazard ratio with 95% CI, models with entropy balancing weights.

Variable	HR (95% CI)	*p*
Grading (G3–4 vs. G1–2)	3.68 (1.79–7.58)	**<0.001**
Gr. 10: N+ vs. N−	4.36 (2.31–8.24)	**<0.001**
Gr. 11: N+ vs. N−	3.44 (1.40–8.42)	**0.007**
Gr. 18: N+ vs. N−	3.28 (1.38–7.81)	**0.007**
LNR > 0 vs. LNR = 0	0.28 (0.04–1.88)	0.191

**Table 8:** Cox multivariate analysis—PFS-DM. Statistically significant values (*p* < 0.05) are bolded. HR = hazard ratio with 95% CI, models with entropy balancing weights.

Variable	HR (95% CI)	*p*
Grading (G3–4 vs. G1–2)	6.40 (3.77–10.87)	**<0.001**
Gr. 10: N+ vs. N−	4.39 (2.08–9.25)	**<0.001**
Gr. 11: N+ vs. N−	3.50 (1.92–6.39)	**<0.001**
Gr. 18: N+ vs. N−	2.94 (1.68–5.15)	**<0.001**
Neoadjuvant chemotherapy (yes vs. no)	0.62 (0.41–0.94)	**0.024**
Location: PB vs. PN	1.47 (0.87–2.48)	0.147
Location: PT vs. PN	0.76 (0.33–1.75)	0.516

## Data Availability

The data are available from the corresponding author upon reasonable request.

## Supplementary Materials

The following supporting information can be downloaded at: https://www.mdpi.com/article/10.3390/cancers18121988/s1, Section S1: Pair Analysis; Table S1: Pairwise comparisons of continuous variables between tumor location groups (PN vs. PB vs. PT) using Dunn’s test with FDR correction–post hoc analyses after a significant global Kruskal–Wallis test. Pairwise comparisons were performed using Dunn’s test only for variables for which the global Kruskal–Wallis test showed statistical significance or trend (*p* < 0.10). Z = standardized rank statistic. *p* = two-sided *p* value. *p* (FDR) = Benjamini–Hochberg-corrected *p* value (false discovery rate). Marked = significant after FDR correction. **** *p* < 0.0001; * *p* < 0.05; n.s. = not statistically significant; ELN = examined lymph nodes—number of all lymph nodes examined during histopathological examination; LNR = lymph node ratio—the ratio of the number of lymph nodes with PDAC metastases to the total number of examined lymph nodes; Table S2: Pairwise comparisons of categorical variables between tumor location groups (PN vs. PB vs. PT) using two-sided Fisher exact test with FDR correction. *p* (Fisher) = two-sided *p*-value from Fisher’s exact test. *p* (FDR) = Benjamini–Hochberg-corrected *p*-value (false discovery rate) per variable. Marked = significant after FDR correction (*p* < 0.05). **** *p* < 0.0001; *** *p* < 0.001; ** *p* < 0.01; * *p* < 0.05; n.s. = not statistically significant; ASA = American Society of Anesthesiologists (anesthetic risk classification); PNI = perineural invasion; VI = vascular invasion; Table S3: Pairwise comparisons of nodal metastasis rates (N+ vs. N−) between tumor location groups (PN vs. PB vs. PT) at three anatomical stations–two-sided Fisher exact test with FDR correction. Pairwise comparisons of metastatic status (≥1 node with metastasis = N+) between the three tumor location groups, performed separately per anatomical station. *p* (Fisher) = two-sided *p* value from Fisher’s exact test. *p* (FDR) = Benjamini–Hochberg correction per station. Marked = significant after FDR correction; Section S2: Correlation Analysis; Table S4. Correlation between the number of ELN (examined lymph nodes) and the number of nodes with metastases per anatomical site–Spearman’s rank coefficient with 95% CI determined by the BCa bootstrap method. ρ = Spearman’s rank correlation coefficient between the ELN and the number of nodes with metastases in a given station. 95% CI = confidence interval determined by the BCa bootstrap method (bias-corrected and accelerated). ρ and *p* marked = statistically significant (*p* < 0.05). Interpretation of correlation strength according to Cohen: |ρ| < 0.10 = negligible; 0.10–0.29 = weak; 0.30–0.49 = moderate; ≥0.50 = strong; Section S3: Equalization of the distribution of confounding variables between location groups using the entropy balancing method—assessment of the balancing quality based on standardized mean differences and effective sample size; Table S5: Diagnostics of group balancing using the entropy balancing method—standardized mean difference (SMD) before and after balancing. SMD = standardized mean difference (max. of 3 pairs of groups). Criterion: |SMD| < 0.10. Method: entropy balancing, moments = 1, estimate ATE, k = 11 covariates. Effective sample size (ESS): PN = 25.7 (49.4%), PB = 49.7 (75.3%), PT = 23.6 (52.4%). Cumulative ESS = 99.0/163 (60.7%); Figure S1: Assessment of the quality of location group alignment using the entropy balancing method–standardized mean differences (SMD) before and after balancing for 11 covariates with the acceptance threshold |SMD| < 0.10; Section S4: Bootstrap optimism-correction (Harrell) of the final models; Table S6: Discrimination of the final (binary-grading) models with bootstrap optimism correction (1000 resamples); Section S5: Proportional-hazards assessment (scaled Schoenfeld residuals) for the final models; Table S7: Proportional-hazards assessment (scaled Schoenfeld residuals; cox.zph) for the final models; Section S6: Internal validation of the final models; Table S8: Internal validation of the final (binary-grading) models: bootstrap optimism-corrected discrimination and calibration (1000 resamples).

## Abbreviations

The following abbreviations are used in this manuscript:

ASA	American Society of Anesthesiologists
ATE	Average Treatment Effect
BR-PDAC	Borderline Pancreatic Ductal Adenocarcinoma
CeT	Celiac Trunk
CHA	Common Hepatic Artery
CI	Confidence Interval
CT	Computed Tomography
DP-S	Distal Pancreatectomy with Splenectomy
ELN	Examined Lymph Nodes
FDR	False Discovery Rate
HR	Hazard Ratio
ISGPD	International Study Group of Pancreatic Surgery
IPW	Inverse Probability Weighting
IQR	Interquartile Range
IVC	Inferior Vena Cava
LA-PDAC	Locally Advanced Pancreatic Ductal Adenocarcinoma
LNR	Lymph Node Ratio
NCCN	National Comprehensive Cancer Network
VI	Vascular Invasion
OS	Overall Survival
PB	Pancreatic Body
PDAC	Pancreatic Ductal Adenocarcinoma
PFS-LR	Progression-Free Survival Local Recurrence
PFS-DM	Progression-Free Survival Distant Metastases
PN	Pancreatic Neck
PNI	Perineural Invasion
PR-PDAC	Potentially Resectable Pancreatic Ductal Adenocarcinoma
PT	Pancreatic Tail
PV	Portal Vein
SMA	Superior Mesenteric Artery
SMV	Superior Mesenteric Vein
