# Supplementary material for: Analysis of the Distribution of Lymph Node Metastases and Their Impact on the Prognosis in Ductal Adenocarcinoma of the Distal Part of the Pancreas—A Single-Center Retrospective Study
Source: Cancers (Basel). 2026 Jun 18;18(12):1988. doi: 10.3390/cancers18121988 (PMC13297199; doi:10.3390/cancers18121988)
Supplement: Supplementary file 1 [file cancers-18-01988-s001.zip › cancers-4358073-supplementary.pdf]

S1. Pair Analysis

Table S1. Pairwise comparisons of continuous variables between tumor location groups (PN vs. PB vs. PT) using Dunn's test with FDR correction–post hoc analyses after a significant global Kruskal–Wallis test. Pairwise comparisons were performed using Dunn's test only for variables for which the global Kruskal–Wallis test showed statistical significance or trend ($p < 0.10$). Z = standardized rank statistic. *p* = two-sided *p* value. *p* (FDR) = Benjamini–Hochberg-corrected p value (false discovery rate). Marked = significant after FDR correction. **** $p < 0.0001$; * $p < 0.05$; n.s. = not statistically significant; ELN = examined lymph nodes—number of all lymph nodes examined during histopathological examination; LNR = lymph node ratio—the ratio of the number of lymph nodes with PDAC metastases to the total number of examined lymph nodes.

**Variable / comparison**	**Pairs of groups**	***Z***	***p***	***p* (FDR)**	
Demographic data					
**Age [years]**					
	PN vs. PB	−0.86	0.387	0.404	n.s.
	PN vs. PT	0.84	0.404	0.404	n.s.
	PB vs. PT	1.71	0.087	0.262	n.s.
**BMI [kg/m²]**					
	PN vs. PB	−1.94	0.052	0.156	n.s.
	PN vs. PT	−1.57	0.117	0.176	n.s.
	PB vs. PT	0.22	0.829	0.829	n.s.
Tumor features					
**Size [cm]**					
	PN vs. PB	0.48	0.631	0.631	n.s.
	PN vs. PT	5.19	**< 0.0001**	**< 0.0001**	****
	PB vs. PT	5.01	**< 0.0001**	**< 0.0001**	****
**CA 19-9 [U/ml]**					
	PN vs. PB	−0.03	0.975	0.975	n.s.
	PN vs. PT	−1.15	0.248	0.373	n.s.
	PB vs. PT	−1.18	0.236	0.373	n.s.
Perioperative variables					
**Time of operation [min]**					
	PN vs. PB	−0.04	0.966	0.966	n.s.
	PN vs. PT	−5.71	**< 0.0001**	**< 0.0001**	****
	PB vs. PT	−5.98	**< 0.0001**	**< 0.0001**	****
**Blood loss [ml]**					
	PN vs. PB	−0.18	0.856	0.856	n.s.
	PN vs. PT	−1.77	0.077	0.138	n.s.
	PB vs. PT	−1.69	0.092	0.138	n.s.
**Time of hospitalization [days]**					
	PN vs. PB	1.54	0.123	0.185	n.s.
	PN vs. PT	−1.26	0.209	0.209	n.s.
	PB vs. PT	−2.80	**0.005**	**0.015**	*

Variable / comparison	Pairs of groups	*Z*	*p*	*p* (FDR)	
Lymph nodes parameters					
**LNR**					
	PN vs. PB	2.04	0.041	0.062	n.s.
	PN vs. PT	2.63	**0.008**	**0.025**	*
	PB vs. PT	0.82	0.414	0.414	n.s.
**Total examined lymph nodes (ELN)**					
	PN vs. PB	2.17	0.030	0.091	n.s.
	PN vs. PT	1.53	0.125	0.188	n.s.
	PB vs. PT	−0.46	0.643	0.643	n.s.
**Total metastatic lymph nodes**					
	PN vs. PB	2.24	**0.025**	**0.038**	*
	PN vs. PT	2.79	**0.005**	**0.016**	*
	PB vs. PT	0.79	0.432	0.432	n.s.

Table S2. Pairwise comparisons of categorical variables between tumor location groups (PN vs. PB vs. PT) using two-sided Fisher exact test with FDR correction. *p* (Fisher) = two-sided p-value from Fisher's exact test. *p* (FDR) = Benjamini–Hochberg-corrected *p*-value (false discovery rate) per variable. Marked = significant after FDR correction ($p < 0.05$). **** $p < 0.0001$; *** $p < 0.001$; ** $p < 0.01$; * $p < 0.05$; n.s. = not statistically significant; ASA = American Society of Anesthesiologists (anesthetic risk classification); PNI = perineural invasion; VI = vascular invasion.

Variable / comparison	Pairs of groups	*p* (Fisher)	*p* (FDR)	
Demographic data				
**Sex**				
	PN vs. PB	1.000	1.000	n.s.
	PN vs. PT	0.840	1.000	n.s.
	PB vs. PT	0.848	1.000	n.s.
**Smoking (yes vs. no)**				
	PN vs. PB	0.063	0.190	n.s.
	PN vs. PT	0.226	0.339	n.s.
	PB vs. PT	0.565	0.565	n.s.
**ASA (I/II/III)**				
	PN vs. PB	0.500	0.750	n.s.
	PN vs. PT	0.874	0.874	n.s.
	PB vs. PT	0.435	0.750	n.s.
Tumor features				
**Resectability (resectable vs. borderline)**				
	PN vs. PB	0.578	1.000	n.s.
	PN vs. PT	0.687	1.000	n.s.
	PB vs. PT	1.000	1.000	n.s.
**Grading (G1–G4)**				

Variable / comparison	Pairs of groups	*p* (Fisher)	*p* (FDR)	
	PN vs. PB	0.980	0.980	n.s.
	PN vs. PT	0.958	0.980	n.s.
	PB vs. PT	0.893	0.980	n.s.
**Staging (I/II/III)**				
	PN vs. PB	0.322	0.322	n.s.
	PN vs. PT	**0.006**	**0.018**	*
	PB vs. PT	0.146	0.219	n.s.
**pT (pT1/pT2/pT3)**				
	PN vs. PB	**0.020**	**0.020**	*
	PN vs. PT	**< 0.001**	**< 0.001**	***
	PB vs. PT	**< 0.001**	**< 0.001**	***
**pN (pN0/pN1/pN2)**				
	PN vs. PB	0.151	0.226	n.s.
	PN vs. PT	**0.005**	**0.016**	*
	PB vs. PT	0.227	0.227	n.s.
Histopathology				
**PNI (yes vs. no)**				
	PN vs. PB	0.035	0.104	n.s.
	PN vs. PT	0.517	0.517	n.s.
	PB vs. PT	0.247	0.370	n.s.
**VI (yes vs. no)**				
	PN vs. PB	0.256	0.619	n.s.
	PN vs. PT	0.413	0.619	n.s.
	PB vs. PT	0.841	0.841	n.s.
Treatment				
**Neoadjuvant chemotherapy (yes vs. no)**				
	PN vs. PB	0.354	0.818	n.s.
	PN vs. PT	0.545	0.818	n.s.
	PB vs. PT	0.847	0.847	n.s.
Complications				
**Clavien–Dindo (0–IV)**				
	PN vs. PB	0.801	0.801	n.s.
	PN vs. PT	0.388	0.582	n.s.
	PB vs. PT	0.221	0.582	n.s.
Status of metastases in lymph node stations				
**Group 10 (N+ vs. N–)**				
	PN vs. PB	0.509	0.509	n.s.
	PN vs. PT	$\mathbf{2.66 \times 10^{-16}}$	$\mathbf{7.98 \times 10^{-16}}$	****
	PB vs. PT	$\mathbf{1.33 \times 10^{-15}}$	$\mathbf{2.00 \times 10^{-15}}$	****
**Group 11 (N+ vs. N–)**				

Variable / comparison	Pairs of groups	*p* (Fisher)	*p* (FDR)	
	PN vs. PB	0.052	0.078	n.s.
	PN vs. PT	0.310	0.310	n.s.
	PB vs. PT	**0.002**	**0.005**	**
**Group 18 (N+ vs. N–)**				
	PN vs. PB	0.095	0.285	n.s.
	PN vs. PT	0.313	0.470	n.s.
	PB vs. PT	0.565	0.565	n.s.

Table S3. Pairwise comparisons of nodal metastasis rates (N+ vs. N−) between tumor location groups (PN vs. PB vs. PT) at three anatomical stations – two-sided Fisher exact test with FDR correction. Pairwise comparisons of metastatic status (≥1 node with metastasis = N+) between the three tumor location groups, performed separately per anatomical station. *p* (Fisher) = two-sided *p* value from Fisher's exact test. *p* (FDR) = Benjamini–Hochberg correction per station. Marked = significant after FDR correction.

Lymph node station / comparison	Pairs of groups	*p* (Fisher)	*p* (FDR)
**Group 10** (*PN 5,8% ǀ PB 10,6% ǀ PT 84,4%*)			
	PN vs. PB	0.509	0.509
	PN vs. PT	**< 0.001**	**< 0.001**
	PB vs. PT	**< 0.001**	**< 0.001**
**Group 11** (*PN 53,8% ǀ PB72,7% ǀ PT 42,2%*)			
	PN vs. PB	0.052	0.078
	PN vs. PT	0.310	0.310
	PB vs. PT	**0.002**	**0.005**
**Group 18** (*PN 42,3% ǀ PB 59,1% ǀ PT 53,3%*)			
	PN vs. PB	0.095	0.285
	PN vs. PT	0.313	0.470
	PB vs. PT	0.565	0.565

S2. Correlation Analysis

Table S4. Correlation between the number of ELN (examined lymph nodes) and the number of nodes with metastases per anatomical site–Spearman's rank coefficient with 95% CI determined by the BCa bootstrap method. ϱ = Spearman's rank correlation coefficient between the ELN and the number of nodes with metastases in a given station. 95% CI = confidence interval determined by the BCa bootstrap method (bias-corrected and accelerated). ϱ and *p* marked = statistically significant ($p < 0.05$). Interpretation of correlation strength according to Cohen: ǀϱǀ < 0.10 = negligible; 0.10–0.29 = weak; 0.30–0.49 = moderate; ≥0.50 = strong.

Lymph node station	*ρ*	*ρ* (95% CI)	*p*	Interpretation
Group 10	**0.20**	0.04–0.35	**0.011**	Weak positive

Group 11	**0.33**	0.19–0.46	**< 0.001**	Moderate positive
Group 18	**0.16**	0.00–0.31	**0.037**	Weak positive
**Total**	**0.16**	0.00–0.32	**0.038**	Weak positive

S3. Equalization of the distribution of confounding variables between location groups using the entropy balancing method—assessment of the balancing quality based on standardized mean differences and effective sample size

The significant differences identified in tumor characteristics between the location groups—in particular, significantly larger tumor size and more advanced pT categories in the pancreatic tail—raised justified concerns that potential differences in survival might reflect not so much the influence of location per se, but rather an asymmetric distribution of variables with documented prognostic relevance. To prevent this source of confounding and enable reliable causal interpretation in subsequent survival models, entropy balancing was used—a nonparametric reweighting method that assigns weights that ensure precise equalization of the moments of covariate distributions between groups, within the framework of the average treatment effect (ATE) estimate. The balancing procedure included 11 covariates selected a priori based on their documented prognostic significance in the PDAC literature: age, sex, BMI, tumor size, grading, pT category, resectability, neoadjuvant chemotherapy, preoperative CA 19-9 level, perineural invasion (PNI), and vascular invasion (VI). The alignment quality diagnostics are presented in Table S5, and its graphical synthesis is presented in Figure S1.

Table S5. Diagnostics of group balancing using the entropy balancing method—standardized mean difference (SMD) before and after balancing. SMD = standardized mean difference (max. of 3 pairs of groups). Criterion: |SMD| < 0.10. Method: entropy balancing, moments = 1, estimate ATE, k = 11 covariates. Effective sample size (ESS): PN = 25.7 (49.4%), PB = 49.7 (75.3%), PT = 23.6 (52.4%). Cumulative ESS = 99.0/163 (60.7%).

**Variable**	**Type**	**SMD before**	**SMD after**	**Rate**
Age	continuous	0.39	0.00	< 0.10
Sex (men)	binary	0.03	0.00	< 0.10
BMI	continuous	0.38	0.00	< 0.10
Size	continuous	1.21	0.00	< 0.10
Grading	continuous	0.12	0.00	< 0.10
pT	continuous	0.79	0.00	< 0.10
Resectability	binary	0.06	0.00	< 0.10
Neoadjuvant chemotherapy	binary	0.10	0.00	< 0.10
CA 19-9	continuous	0.27	0.00	< 0.10
PNI	binary	0.18	0.00	< 0.10
VI	binary	0.11	0.00	< 0.10

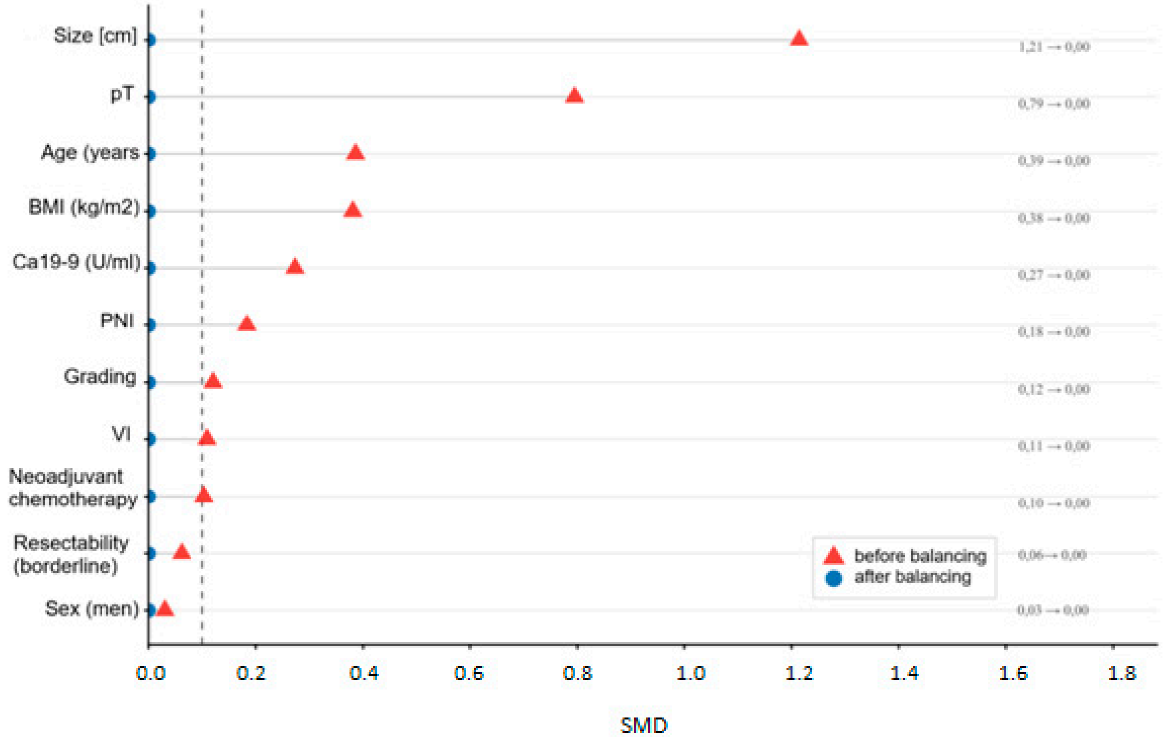

Figure S1. Assessment of the quality of location group alignment using the entropy balancing method—standardized mean differences (SMD) before and after balancing for 11 covariates with the acceptance threshold |SMD| < 0.10.

Before applying entropy balancing, the imbalance profile between the site groups was significant and heterogeneous. The standardized mean difference (SMD), reported as the maximum across all three pairs of groups, exceeded the acceptable threshold of |SMD| < 0.10 for several key variables. The most imbalanced variable was tumor size (SMD = 1.21), which corresponds to a difference exceeding one standard deviation and is a consequence of the previously described tumor size gradient growing toward the pancreatic tail. The pT category (SMD = 0.79), which is a derivative of both tumor size and local stage, ranked second. Other variables with a moderate degree of imbalance included age (SMD = 0.39), BMI (SMD = 0.38), and preoperative CA 19-9 level (SMD = 0.27) – three parameters that did not reach statistical significance in the global tests in the descriptive analysis, but whose combined confounding potential required correction, especially in the context of multivariate models.

Among the binary variables, PNI (SMD = 0.18), VI (SMD = 0.11), and neoadjuvant chemotherapy (SMD = 0.10) showed imbalances bordering on or slightly above the acceptance threshold. The only variable that met the imbalance criterion even before balancing was sex (SMD = 0.03), which corresponds to the lack of any intergroup differences.

After applying entropy balancing weights, the distribution of all 11 covariates was completely equalized: the SMD for each variable reached 0.00, indicating perfect balance of the first moments of the distributions and elimination of any measurable confounding. This result represents a significant added value compared to classical propensity score matching (PSM), which requires model specification adjustments and does not guarantee exact balancing, only an approximation. In this analysis, entropy balancing provided an accurate, single-step solution, eliminating the need to verify multiple alternative propensity model specifications.

An issue that requires transparent discussion is the effective sample size (ESS), which inevitably decreases as a result of imposing unequal weights. The total ESS was 99.0 of 163 observations (60.7%), with the degree of reduction unevenly distributed between groups: the core retained the highest effective number of observations, 49.7 (75.3% of the initial n), reflecting its intermediate position in the spectrum of clinicopathological characteristics and the resulting reduced need for reweighting. PN and PT, the extreme groups in terms of tumor profile, underwent more intensive correction, resulting in ESS of 25.7 (49.4%) and 23.6 (52.4%), respectively. Reducing the ESS by approximately 40% compared to the full sample represents an inherent trade-off for any weighting technique: the gain in confounding is accompanied by a loss of statistical precision, expressed through

wider confidence intervals and higher standard errors. However, the adopted ESS level was deemed acceptable for two reasons: first, the effective sample size remains sufficient for stable parameter estimation in Cox models with the planned number of covariates; second, the alternative approach—no balancing—would expose the results to potentially larger errors resulting from uncorrected confounding, especially for variables with SMD > 0.50 (tumor size, pT), whose impact on survival is well documented.

The entropy balancing procedure achieved its initial goal—full equalization of the 11 covariates between the three nonrandomized tumor site groups, while maintaining an effective sample size that allowed for further multivariate modeling. The resulting weights form the foundation for all subsequent survival analyses: Kaplan–Meier estimation (Manuscript, Section 3.4) and univariate and multivariate Cox models (Manuscript, Sections 3.5–3.6), ensuring that the location effects observed in these analyses reflect – within the limits of measurable variables – the true effect of tumor position and not an artifact of baseline differences between groups.

S4. Bootstrap optimism-correction (Harrell) of the final models

Bootstrap optimism-correction (Harrell) of the final models showed negligible shrinkage in discrimination: OS C = 0.836 apparent, 0.831 corrected (optimism 0.005); PFS-LR C = 0.810 apparent, 0.803 corrected (optimism 0.006); PFS-DM C = 0.837 apparent, 0.828 corrected (optimism 0.009) (Table S6). An optimism of well under 0.01 is direct evidence that the parsimonious models are not materially overfit.

Table S6. Discrimination of the final (binary-grading) models with bootstrap optimism correction (1000 resamples).

Endpoint	Apparent C	Optimism	Optimism-corrected C
OS	0.836	0.005	0.831
PFS-LR	0.810	0.006	0.803
PFS-DM	0.837	0.009	0.828

Conclusion: Optimism below 0.01 indicates the parsimonious models are not materially overfit.

S5. Proportional-hazards assessment (scaled Schoenfeld residuals) for the final models.

Proportional hazards were assessed for every covariate and globally using scaled Schoenfeld residuals (the cox.zph procedure).

Table S7. Proportional-hazards assessment (scaled Schoenfeld residuals; cox.zph) for the final models.

Model	Grading (p)	Stations 10/11/18 (p)	Neoadjuvant (p)	Global (p)
OS	0.004 *	0.30 / 0.76 / 0.34	0.18	0.038
PFS-LR	0.45	0.39 / 0.10 / 0.19	–	0.46
PFS-DM	0.008 *	0.50 / 0.87 / 0.10	0.28	0.085

The nodal-station effects – the scientific core of the paper – satisfy proportional hazards in all models, as does neoadjuvant chemotherapy. The grading effect is non-proportional for OS and PFS-DM. Rather than discard it, we now interpret the grading hazard ratio explicitly as a time-averaged effect over the follow-up period and note the violation in the text; as a directional sensitivity check, a model with a grade-by-time interaction confirms that the grading effect attenuates with time but remains protective-direction-consistent and does not alter the nodal-station estimates.

S6. **Internal validation of the final models.**

Calibration was assessed by the bootstrap optimism-corrected calibration slope, which measures the shrinkage required to offset over-optimistic coefficients; a value near unity denotes agreement between predicted and observed risk. We note that an apparent (in-sample) concordance index with an asymptotic confidence interval would not, on its own, constitute internal validation, since it does not correct for optimism – hence the resampling approach adopted here.

The results, in Table S8, are reassuring: optimism in discrimination is negligible (≤ 0.008) and the optimism-corrected calibration slope is close to unity at every endpoint (0.92–0.95), indicating well-calibrated models with only minimal over-fitting.

Table S8. Internal validation of the final (binary-grading) models: bootstrap optimism-corrected discrimination and calibration (1000 resamples).

Endpoint	Apparent C	Optimism-corrected C	Optimism-corrected calibration slope
OS	0.836	0.832	0.95
PFS-LR	0.810	0.802	0.93
PFS-DM	0.837	0.829	0.92

Note. Discrimination is Harrell's C; optimism is estimated by repeating model fitting in each bootstrap resample and evaluating it on the original data. The calibration slope is the optimism-corrected coefficient of the linear predictor; a value of 1 denotes perfect calibration.

The C-statistics correspond to those in Table S6
